# Efficient ReML inference in variance component mixed models using a Min-Max algorithm

**DOI:** 10.1371/journal.pcbi.1009659

**Published:** 2022-01-24

**Authors:** Fabien Laporte, Alain Charcosset, Tristan Mary-Huard

**Affiliations:** 1 Université Paris-Saclay, INRAE, CNRS, AgroParisTech, Génétique Quantitative et Evolution - Le Moulon, Gif-sur-Yvette, France; 2 INRAE, AgroParisTech, Université Paris-Saclay, MIA-Paris, Paris, France; Princeton University, UNITED STATES

## Abstract

Since their introduction in the 50’s, variance component mixed models have been widely used in many application fields. In this context, ReML estimation is by far the most popular procedure to infer the variance components of the model. Although many implementations of the ReML procedure are readily available, there is still need for computational improvements due to the ever-increasing size of the datasets to be handled, and to the complexity of the models to be adjusted. In this paper, we present a Min-Max (MM) algorithm for ReML inference and combine it with several speed-up procedures. The ReML MM algorithm we present is compared to 5 state-of-the-art publicly available algorithms used in statistical genetics. The computational performance of the different algorithms are evaluated on several datasets representing different plant breeding experimental designs. The MM algorithm ranks among the top 2 methods in almost all settings and is more versatile than many of its competitors. The MM algorithm is a promising alternative to the classical AI-ReML algorithm in the context of variance component mixed models. It is available in the MM4LMM R-package.

## Author summary

Mixed models have been a cornerstone of the quantitative genetics methodology for decades. Due to the growing size of datasets, their associated computational cost is a major burden, particularly in genome-wide association studies that routinely address Millions of markers, requiring as many mixed models to be fitted. In the particular case of a 2-variance component mixed models efficient procedures such as FaST-LMM or GEMMA have been developed to analyze panels with tens of thousands of individuals. However, there is room for improvement in cases where the computational burden is due to the number of variance components in the model rather than the panel size, a classical situation in plant genetics where several variance components are required to handle various polygenic effects. We consider a “MM” (Min-Max) algorithm as an alternative to the by-default AI (Average Information) algorithm used to perform inference in mixed models. The MM algorithm can be combined to the classical tricks used to accelerate the inference process (e.g. simultaneous orthogonalization or squared iterative acceleration methods). The MM procedure is implemented in an MM4LMM package, and is competitive compared to classical algorithms used in plant genetics. This package should help geneticists handling more complex models to analyze their data than today.

This is a *PLOS Computational Biology* Methods paper.

## Introduction

Since their formal introduction in the early 50’s [1, 2], mixed models have become an indispensable tool of modern statistics. They have been successfully used in many application fields [3] to model data with multiple sources of biological or technical variations. Starting with the work of [4] and [5], mixed models have been a favored methodology in quantitative genetics, and are still widely used in the context of Genome-Wide Association Studies (GWAS) and Genomic Selection (GS).

With the development of high throughput technologies, a special care has been dedicated to the development of efficient algorithmic procedures for the inference of mixed models [6–9]. This is illustrated by the availability of many tools/softwares that either perform inference in a mixed model including many (fixed and random) effects on large datasets, or alternatively that efficiently fit hundreds of thousands of mixed models with a limited number of variance components. Many of these tools were developed in the context of animal or human quantitative genetics, or single environment plant studies, where a popular strategy for GWAS analysis is the one presented in [10] that requires the fitting of a simple 2-component mixed model per marker, a favorable case for which efficient algorithms exist that allow the analysis of very large panels with 10^4^ − 10^5^ individuals genotyped at 10^6^ markers [7, 8].

In the context of plant breeding, panels are often of moderate size, including a few hundreds/thousands of individuals only due to experimental/cost constraints. At the same time, the mixed models used for the statistical analysis may be more complex that the ones used in human genetics, since the modeling should account for the specificities of the experimental design (e.g. when a multi-site experiment involving the same varieties in different environments has been carried out) or of the crossing design (e.g. when considering hybrids obtained from parental lines belonging to different populations). One then aims at choosing among the many algorithmic procedures the one that is best suited to cope with these specific features.

From a technical point of view, the Average Information (AI) algorithm [11, 12] has become the reference algorithm for Restricted Maximum Likelihood (ReML) estimation. It has been implemented in many packages [9, 13, 14] and different contexts, the practical implementation being sometimes slightly different from a package to another (see the Methods section). Although in some very specific cases alternative algorithms may be more computationally efficient, it is considered as a reference due to its versatility and its competitive computational performance.

In this article we present a Min-Max (MM) algorithm for the ReML inference in Gaussian Variance Component (VC) mixed model. MM algorithms have been previously described [15], and applied to ML inference in mixed models [16]. We first present here the full derivation of the MM procedure applied to ReML estimation, along with the way it can be combined with some classical computational tricks to significantly speed-up the initial procedure. We then provide a thorough benchmark comparison to illustrate that the proposed MM algorithm can compete with several state-of-the-art algorithms that are widely used in quantitative genetics to perform ReML estimation, including BOLT-LMM [17], FaST-LMM [7], gaston [9], GEMMA [18] and GridLMM [19]. In our study algorithms are compared on different plant breeding scenarios, with a focus for those that may require numerous runs of the model (e.g. GWAS). The study reveals that differences in terms of precision may be marginal between exact ReML procedures, but differences in computational performance may be important. In many scenarios the MM algorithm proves to be competitive with respect to its competitors.

## Results

### Setting

We considered several procedures corresponding to different implementations of the optimization algorithms described in the Methods section. Our candidate procedure, called hereafter MM4LMM, corresponds to the implementation of the Min-Max algorithm. MM4LMM is compared to the following 3 state-of-the-art procedures:

⋆ gaston [9] (version 1.5.7) is an R package that implements the AI algorithm. When *K* = 2 gaston performs ReML estimation using Newton method.
⋆ FaST-LMM [7] (C version 2014) fits mixed models with two variance components only, using the joint orthogonalization and profiling tricks.
⋆ GEMMA [18] (version 0.98.1) fits mixed models using the AI algorithm for variance component estimation, combined with the joint orthogonalization trick when applied to a two variance component model.

All aforementioned algorithms aim at optimizing the restricted maximum likelihood (6). Additionally, we considered Grid-LMM [19] that performs an approximate ReML optimization over a grid of candidate variance values, and BOLT-LMM that performs GWAS using a Gaussian mixture modeling of SNP effects (see [17] for details).

In order to compare the different algorithms in terms of precision of variance parameter estimation, one needs a reference procedure that provides the “true” values of the ReML estimates. In the present context we considered the optimizeLmer function of the lme4 [20] R package which performs inference using the BOBYQA algorithm [21]. This algorithm was used as the reference procedure, lme4 being one of the most popular (and consequently one of the most debugged) R package to fit mixed models.

We focus here on the application of mixed models to statistical plant genetics, with two specific application cases in mind: genome-wide association study (GWAS) and variance component analysis (VCA). In GWAS, the goal is to identify markers that are significantly associated with phenotypic variation. The relationship between a given marker and the phenotype needs to be tested in a model that accounts for both the marker effect and background genetic effects specific to each individual.

In classical human and animal studies and also simple plant experiments (one trait in one environment, single population), it is classical to adjust LMM with two variance components (genetic background and residual). Therefore, many softwares exist to fit LMM with only two variance components. However, a number of plant and animal genetic studies address phenotypic data of hybrids (crosses in animals) between different populations, referred to in plants as heterotic groups (e.g. flint and dent for maize genetics). Background genetic effects involve in this case more than one source of variation, at least four when considering the additive variation contributed by each group, the interaction between the two groups and the error term.

A further characteristic of plant experiments is that phenotyping of varieties is generally conducted in multiple environments. These different environments generally reveal different magnitudes of genetic and error variations and complex genotype x environment patterns. In this case, algorithms have to be flexible regarding the high number of possible random effects considered within the LMM.

Most GWAS algorithms fit mixed model including two or more variance components for each tested marker, meaning that computational efficiency is of major importance. Alternatively, some algorithms build on an approximate procedure where only the parameters associated to the fixed part of the model are updated for each marker (e.g. EMMAX or the approximate version of FaST-LMM, see [7, 22]), resulting in a significant computational speed-up. However, one should be sure that such speed-up approximations in the optimization algorithm yield accurate *p*-values.

### Algorithm comparison

The three datasets presented in the next sections were selected as representative of classical GWAS/GS/VCA panels in plant quantitative genetics, both in terms of scale (numbers of trials, individuals per trials and replicates per individuals) and complexity (lines or hybrids, need for gene×environment interaction in the statistical modeling…). All datasets are publicly available, see the Data Availability section. Also we stress out that the models considered for each dataset were selected in order to illustrate the many different aspects that may impact the computational performance of the algorithms to be compared rather than providing general guidelines about statistical modeling in quantitative genetics.

All algorithms were run and compared on a server using a Intel(R) Xeon(R) CPU E5–2420 0 @ 1.90GHz processor, applying their by-default configuration settings. Each dataset was analyzed using the same number of cores and memory setting for all algorithms.

#### Two variance components

**Dataset**. We consider the CornFed Flint Association panel—named Flint hereafter [23]. It consists in 259 maize lines of the Flint heterotic group, genotyped at 39,076 biallelic markers (after quality control). These hybrids were evaluated in an 11 location network. In the following GWAS we considered least square means of the individual phenotypes as the studied trait as in the original publication. We present here the results for two phenotypes, DM_Y_Flo and Tass, the results being similar for the remaining ones.

**Statistical analysis**. For each marker *ℓ* and for a given trait, the analysis was performed using the following model:

$$\begin{array}{ccc} & & Y=\mu +X_{\ell }\beta_{\ell }+U+E\;,\\ & & U∼N\left(0,\sigma_{G}^{2}K\right),\;\;E∼N\left(0,\sigma_{E}^{2}I\right)\;\;\text{and}\;U⊥E, \end{array}$$

with *Y* the vector of phenotypes, *X*_*ℓ*_ the vector corresponding to the number of copies of allele 1 present at marker *ℓ*, *β*_*ℓ*_ the effect associated with allele 1, *U* the random effect accounting for the genetic background, and *E* the error vector. *K* is the matrix of kinship between lines. To identify markers associated to phenotypic variation the null hypothesis *H*_0_: {*β*_*ℓ*_ = 0} was tested using a Wald test procedure (see S1 Appendix for details).

**Computational time**. The computational times corresponding to the whole genome analysis are displayed in Table 1. Recall that lme4 represents the reference in terms of inference performance (i.e. sharp estimation of parameters), but is not expected to perform well in terms of computational time, being not optimized for GWAS analysis. Although all methods achieve the GWAS analysis in less than 30s, a factor of at least 10 may be observed between the most efficient (gaston) and the less efficient (FastLMM or BOLT-LMM) methods (excluding lme4). There is no gain in using GridLMM in this context since all exact methods are using the simultaneous orthogonalization trick, making the inference very efficient. One also observes that the computational time of BOLT-LMM is quite unstable from one trait to another.

**Table 1:** Computational time (in sec.) associated to the different algorithms for the complete GWAS analysis of the Flint dataset, trait by trait.

	gaston	MM4LMM	FaST-LMM	GEMMA	BOLT-LMM	GridLMM	lme4
DM_Y	3	6	28	15	12	9	12886
Tass	5	17	28	15	325	5	34852

**QTL detection**. In order to check the precision of the methods, we applied a full marker identification procedure accounting for multiple testing with each algorithm. A Bonferroni correction using *M*_*eff*_ as the number of tests was performed, with *M*_*eff*_ being the effective number of tests as estimated using the Gao procedure [24]. For the present application one obtains *M*_*eff*_ = 3, 527.

We found the GWAS results to be very similar across all algorithms except BOLT-LMM. For the other algorithms all GWAS analyzes led to the same list of markers (provided in Table 2), and the correlation between the different lists of (log-transformed) p-values were found to be higher than 0.99. The correlation observed between BOLT-LMM and the other methods is 0.97, leading to a different list of significant markers. This could be expected since BOLT-LMM does not rely on the same modeling than the other algorithms.

Several approximate approaches have been proposed in order to further reduce the computational burden. In gaston a score test procedure is provided. FaSTLMM also comes with an approximate version of the test procedure, where the variance components are estimated only once without further refitting of the restricted maximum likelihood for each marker. Although such strategies speed up the computational performance, they result in p-values that are poorly estimated, and possibly to different lists of significant markers (see S1 Code: TwoVarianceComponents/Rmd/SupplementaryResults_TVC.html). Alternatively, it has been advocated to perform GWAS by considering each chromosome separately for the variance component estimation, using a kinship matrix estimated on markers that do not belong to the candidate chromosome under study [25]. Applying this strategy led to an increase of power (i.e. smaller p-values for the best candidates) for the approximate approaches. However in the present example this gain in power did not increase the number of markers detected by the approximate methods up to that detected by the exact ones. Additionally, note that exact procedures can also be combined to this “leave-one-chromosome-out” approach, (see also S1 Code: TwoVarianceComponents/Rmd/SupplementaryResults_TVC.html).

**Table 2:** −log_10_(p-value) of markers detected by all exact algorithms for DMY_Flo.

Marker	−log_10_(*pval*)
SYN10537	5.61
SYN10528	5.61
PZE-101030022	5.07
PZE-101123079	4.87
SYN13856	5.19

List of significant markers at a nominal level of 5% (Gao correction for multiple testing).

In the next two sections BOLT-LMM and FaST-LMM are not considered since they do not handle models with more than 2 variance components.

#### Four variance components

**Dataset**. The maize dataset (called Factorial in the following) consists in hybrids derived from an incomplete factorial crossing design between flint and dent lines [26]. A total of *n*_*D*_ = 123 parental dent lines and *n*_*F*_ = 86 parental flint lines were crossed to obtain *n*_*H*_ = 1, 254 hybrids. Parental lines were genotyped at 32,486 markers. Two phenotypic traits were quantified: grain yield (GY) and grain moisture (GM). We present the results obtained on GM. The association study was performed using the following model: at a marker *ℓ* and for a given trait, one has:

$$\begin{array}{c} \begin{array}{c} Y=1\mu +X_{\ell }\beta_{\ell }+Z_{F}G_{F}+Z_{D}G_{D}+Z_{H}G_{H}+E\\ G_{F}∼N\left(0,\sigma_{F}^{2}K_{F}\right),\;G_{D}∼N\left(0,\sigma_{D}^{2}K_{D}\right)\\ G_{H}∼N\left(0,\sigma_{H}^{2}\Phi \right),\;E∼N\left(0,\sigma_{E}^{2}I\right)\\ G_{F}⊥G_{D}⊥G_{H}⊥E \end{array} \end{array}$$ (1)

with *Y* is the vector of phenotype, *X*_*ℓ*_ is the vector corresponding to the number of copies of allele 1 present at marker *ℓ*, *β*_*ℓ*_ the effect associated with the allele 1, *G*_*F*_, *G*_*D*_ and *G*_*H*_ are the random polygenic effects corresponding to the flint parent, the dent parent and their specific interaction, respectively, and *Z*_*F*_, *Z*_*D*_ and *Z*_*H*_ are the associated incidence matrices. Correlation matrices *K*_*F*_ and *K*_*D*_ correspond to the kinship matrices between the dent (resp. flint) parental lines. Matrix *Φ* corresponds to the double relatedness matrix between hybrids, of general term

$$\begin{array}{c} {\hat{\Phi }}_{h,h^{\prime }}={\left({\hat{K}}_{F}\right)}_{f,f^{\prime }}\times {\left({\hat{K}}_{D}\right)}_{d,d^{\prime }} \end{array}$$

where *h* (resp. *h*′) is the hybrid resulting from the crossing between the flint and dent lines *f* and *d* (resp. *f*′ and *d*′). Lastly, *E* is the error vector.

**Fig 1:**
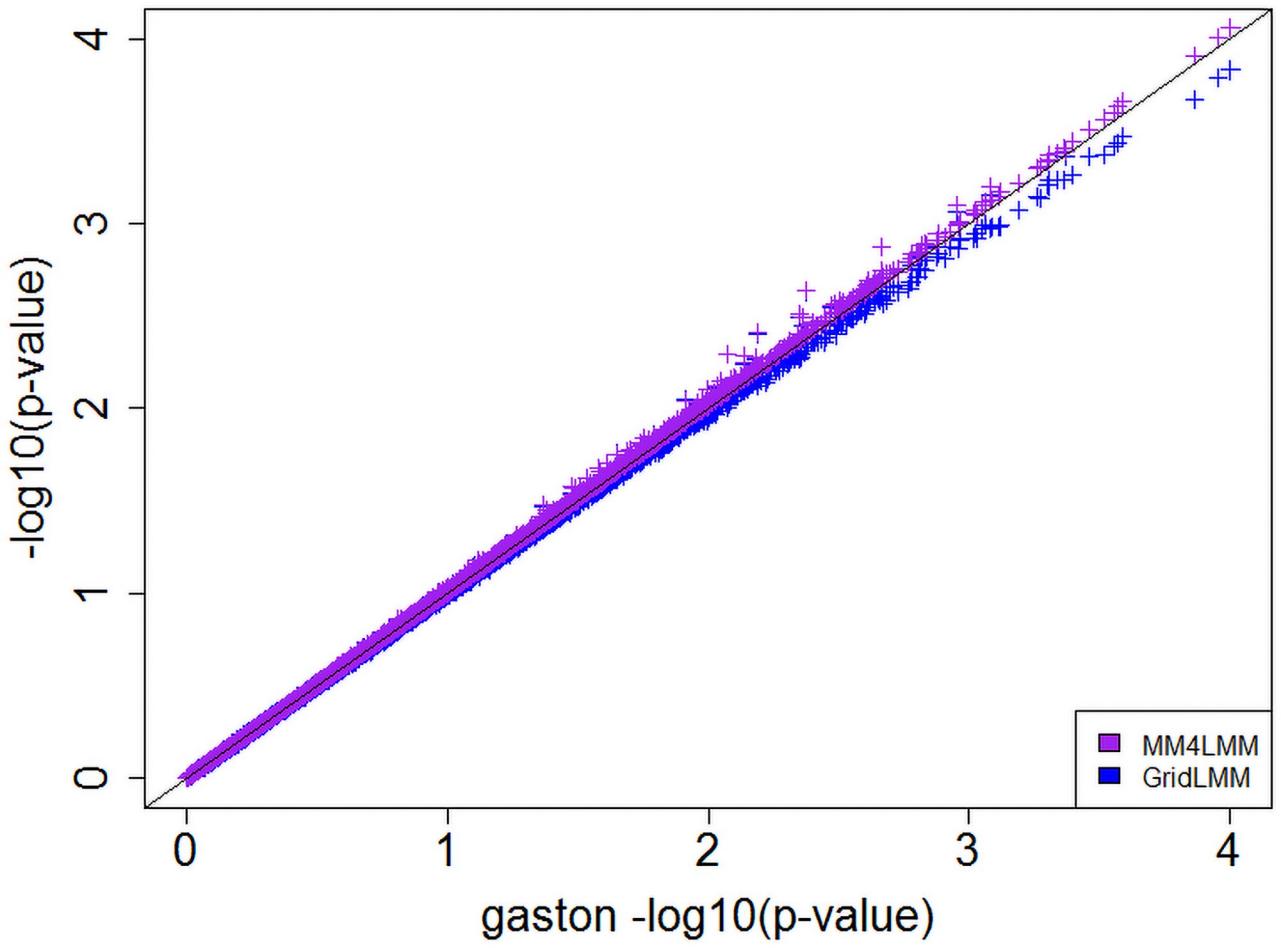
Log-transformed p-values concordance between gaston and GridLMM, and gaston and MM4LMM.

**Algorithm performances**. The analysis of the Factorial dataset required 11.25h with gaston, 2.5h with MM4LMM and 107 seconds with GridLMM. The performance of GEMMA is not reported since it exceeded 20h. In terms of p-values the two exact methods led to very similar results (correlations between −log_10_(p-values) series >0.999). The correlation between any exact method and GridLMM was found to be of the same order but with p-values being bigger for GridLMM compared with exact methods, see Fig 1. Although in the present example no marker was declared significant whatever the method, the p-value inflation could result in a slight loss power in some applications.

Importantly, the model considered for the analysis only assumed an additive effect for the marker. However in most cases the analysis of a hybrid panel requires one to account for both an additive and a dominance fixed effects. Including a dominance effect is possible when using MM4LMM, GEMMA and gaston, but not when using GridLMM that requires the marker effect to be included in the model through a single numeric incidence vector. This constitutes a significant limitation for the use of GridLMM applied to GWAS in the context of plant or animal genetics.

#### More than 4 variance components

**Dataset**. The dataset (called the NAM dataset hereafter) is constituted of *n*_*H*_ = 951 maize hybrids derived from an incomplete factorial crossing design between *n*_*D*_ = 875 dent lines and *n*_*F*_ = 883 flint lines [27]. All hybrids were evaluated for 4 phenotypes (i.e. response variable). Here we focus on the Dry Matter Yield (hereafter DMY), the results obtained with the other traits being very similar. Hybrids were evaluated in 8 different trials performed in two countries, with a number of measurements per hybrid in a trial going from 0 to 2, most hybrids being measured once. The number of measurements per trial goes from 896 to 1001, the total number of measurements being 7,725. The goal of the study was to evaluate the contribution of the 2 parental populations (dent and flint) to the phenotypic variability.

**Statistical analysis**. Two different strategies were considered for the statistical analysis. The first strategy consisted in a 2-step analysis. In step 1 a first model was fitted to correct the phenotypic data for field effects—see S2 Appendix for details. In a second step a Variance Component Analysis was performed using the following model:

$$\begin{array}{c} \begin{array}{c} Y=1\mu +X_{T}\beta_{T}+Z_{F}G_{F}+Z_{D}G_{D}+Z_{H}G_{H}+E\\ G_{F}∼N\left(0,\sigma_{F}^{2}K_{F}\right),\;G_{D}∼N\left(0,\sigma_{D}^{2}K_{D}\right)\\ G_{H}∼N\left(0,\sigma_{H}^{2}\Phi \right),\;E∼N\left(0,\sigma_{E}^{2}I\right)\\ G_{F}⊥G_{D}⊥G_{H}⊥E \end{array} \end{array}$$ (2)

where *Y* here stands for the corrected phenotypes obtained in step 1. Here *β*_*T*_ is the the vector of trial fixed effects and *X*_*T*_ is the associated incidence matrix, the other terms being defined as in Eq (1).

The second strategy consisted in performing a one-step analysis on the whole dataset, including all trials in a single analysis. The model has to account for both genetics and trial/field effects, but also for gene×environment interactions, a feature that is relevant whenever trials are expected to be diverse (in terms of environmental conditions) and genetic effects sensitive to the environment. The random interaction terms are assumed to have specific variances in each trial, leading to

$$\begin{array}{ccc} & Y= & 1\mu +X_{T}\beta_{T}+Z_{F}G_{F}+Z_{D}G_{D}+Z_{H}G_{H}\\ & & +\sum_{k=1}^{N_{T}}[Z_{FT_{k}}G_{FT_{k}}+Z_{DT_{k}}G_{DT_{k}}+Z_{HT_{k}}G_{HT_{k}}\\ & & +Z_{rowT_{k}}G_{rowT_{k}}+Z_{colT_{k}}G_{colT_{k}}+Z_{T_{k}}E_{k}]\\ & G_{FT_{k}} & ∼N\left(0,\sigma_{FT_{k}}^{2}K_{F}\right),\;G_{DT_{k}}∼N\left(0,\sigma_{DT_{k}}^{2}K_{D}\right)\\ & G_{HT_{k}} & ∼N\left(0,\sigma_{HT_{k}}^{2}\Phi \right),\;G_{rowT_{k}}∼N\left(0,I_{n_{r_{k}}}\right)\\ & G_{colT_{k}} & ∼N\left(0,I_{n_{c_{k}}}\right)\;E_{k}∼N\left(0,\sigma_{ET}^{2}I_{n_{k}}\right)\\ & G_{F}⊥ & G_{D}⊥G_{H}⊥G_{FT_{k}}⊥G_{DT_{k^{\prime }}}⊥G_{HT_{k^{\prime \prime }}}⊥E_{k^{\prime \prime \prime }}, \end{array}$$ (3)

with *N*_*T*_ the number of trials, $G_{FT_{k}}$ (resp. $G_{DT_{k}}$ and $G_{HT_{k}}$) the polygenic effect associated to the flint lines (resp. dent lines and flint-dent line interaction) within trial *k*, $Z_{FT_{k}}$, $Z_{DT_{k}}$ and $Z_{HT_{k}}$ the associated incidence matrices, $G_{rowT_{k}}$ and $G_{colT_{k}}$ are row and column effects within trial *k*, $Z_{rowT_{k}}$ and $Z_{colT_{k}}$ the associated incidence matrices, $n_{r_{k}}$ and $n_{c_{k}}$ are the numbers of row and column within trial *k* and *E*_*k*_ the residual effect within the trial *k*, *n*_*k*_ the number of hybrids within trial *k* and $Z_{T_{k}}$ the incidence matrix associated to *E*_*k*_. Note that residual variances are also assumed to be specific to each trial.

In multi-trial analyzes different factors may impact the computational efficiency of the inference algorithm, including the number of observations, the number of trials, the computational tricks that may be implemented and the number of variance components. In order to quantify the effect of these factors on the different algorithms considered here, we first considered simulated data mimicking a subsample of the full dataset. Synthetic phenotypes were simulated based on the observed genotypic data and the experimental design of the NAM dataset. More precisely, we subsampled sets of hybrids from the NAM dataset and then simulated the phenotype based on Model (2), using the observed kinship matrices and considering no fixed effects (i.e. all observations have a null mean). The computational performance of the 4 algorithms are presented on both the simulated and the NAM data, analyzed using either Model (2) or (3). Note that Model (3) has 44 components when assuming a common error variance, and 51 otherwise, making the model fitting significantly more involving than the previous analyses. Consequently we considered variance component analysis rather than association analysis.

**Estimation**. In terms of variance estimation, all algorithms yielded the same results when applied to the complete NAM dataset, using Model (2). The table of the variance component estimates is given in S3 Appendix. Similar conclusions were obtained when considering other phenotypic traits (results not shown).

**Fig 2:**
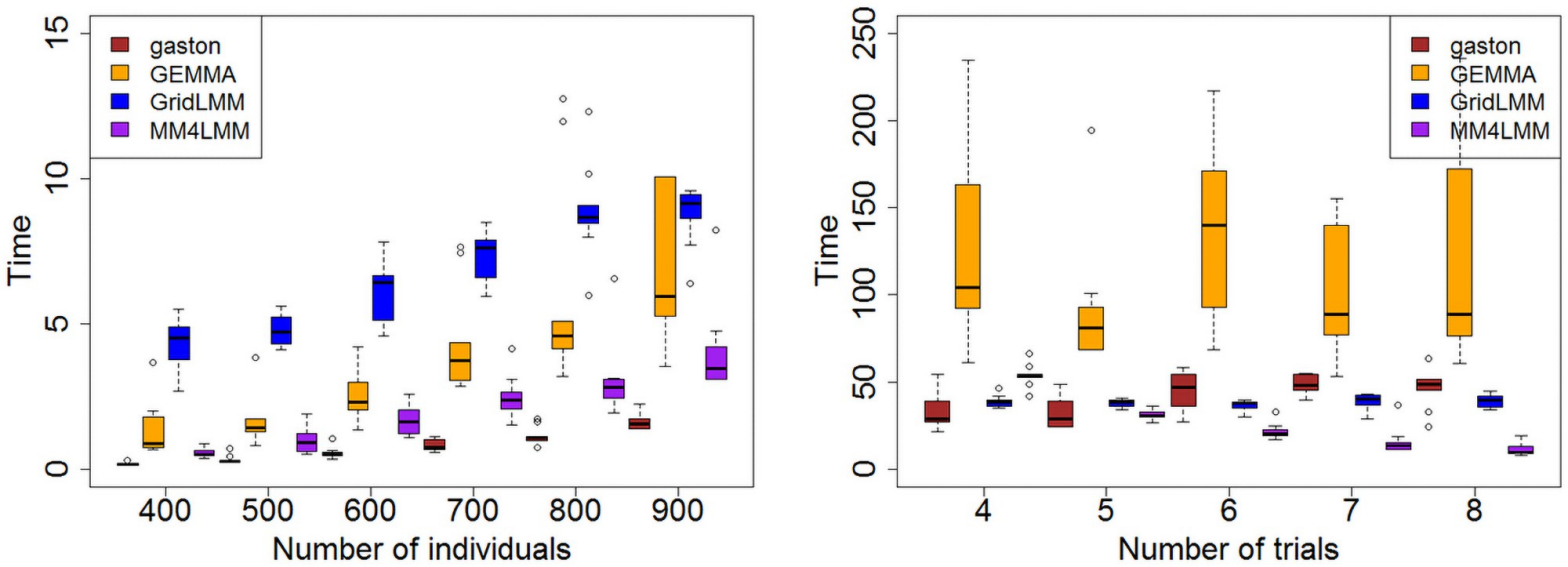
Computational time for variance component analysis with simulated data. Computational time of the algorithms with respect to the number of observations (left) and the number of trials (right).

**Computational time**. We investigated how the number of observations impacts the computational performance of the different algorithms. We first considered a “one-trial” simulation setting where *n* = 400, 500, …, 900 hybrids were randomly selected from the 951 available ones and phenotypes were generated as described in the previous paragraph. This process was repeated 10 times. The data were then analyzed using Model (2). The results are displayed in Fig 2 (left). As expected the computational time of all procedures increases with *n*, with gaston and MM4LMM being the top two algorithms. Compared to the Hybrid data application the lower performance of GridLMM comes from the fact that here only a single model is fitted.

In a second setting we investigated the impact of the number of trials on the computational performance of the different algorithms. To this end, we fixed the total number of observations at *n* = 2400, and randomly sampled hybrids in *n*_*T*_ = 4, …, 8 trials (balancing the contribution of the different trials). For each number of trials, the simulation process was repeated 10 times and Model (2) was used for the analysis. Results are presented in Fig 2 (right). Apart from MM4LMM, all algorithms are insensitive to *n*_*T*_: as soon as the number of variance components in the model does not depend on *n*_*T*_ the algorithms scale with *n* (which is fixed here) only. The behavior of MM4LMM differs from the other algorithms because MM4LMM automatically selects whether the MME trick (described in the Methods section) should be used or not. In the present setting one can show that the algorithmic complexity of the MM algorithm is *O*(*n*^3^ + *K* × *n*^2^ + *p*^3^) whereas the one of MM combined with the MME trick is $O({\left(n_{F}+n_{D}+n_{H}+p\right)}^{3}+\left(n_{F}^{2}+n_{D}^{2}+n_{H}^{2}+\sum_{k=1}^{K}n_{k}\right)+p^{3})$, where *p* = *rank*(*X*). Here quantities *n*_*F*_, *n*_*D*_ and *n*_*H*_ decrease with increasing values of *n*_*T*_, and the computational time of MM4LMM decreases accordingly. Consequently, depending on the balance between the number of random effects and of measurements, the MME trick may be beneficial (e.g. when all trials are considered) or detrimental (eg when only 2 or 4 trials are included in the analysis).

Although the previous simulated settings allow one to disentangle the effect of the number of observations and the number of trials, we considered a more realistic setting where both numbers increase together, i.e. a setting where the number of slots in a trial does not depend on *n*_*T*_. Here we used the real NAM data, built intermediate versions of the complete dataset by selecting subsets of 2, 4 or 6 trials among the 8 available ones and analyzed these subsets with Model (2). Table 3 displays the computational time associated with each algorithm. One observes that differences in terms of performance may be important, as quantified using the ratio between the worst and the best computational time obtained (last column), with no single algorithm being uniformly the most efficient. Note that the Bayesian estimation procedure implemented in the MCMCglmm package [28] was also considered for this analysis. Results were similar in terms of parameter estimation but are not reported here due to prohibitive computational costs (>24h for a single 2-trial analysis).

**Table 3:** Computational time (in sec.) associated to the analysis of different subsamples of trials of the NAM dataset, using Model (2). Bold numbers correspond to the best performance.

Nb Trials	Avg Nb Obs	gaston		MM4LMM		GEMMA		GridLMM		Ratio
		mean	sd	mean	sd	mean	sd	mean	sd	
2	1,931.25	**16.28**	4.43	42.15	7.05	58.35	16.48	23.02	4.45	3.6
4	3,862.50	170.44	24.15	288.08	44.62	386.03	30.02	**106.74**	14.28	3.6
6	5,793.75	659.35	72.58	**308.11**	25.87	1244.19	152.15	326.58	37.15	4.0
8	7,725	1786.87		**333.15**		3100.94		792.59		9.3

We then considered the analysis of all trials using Model (3). In this context the use of the MME trick would be detrimental since the cumulative size of the correlation matrices may become significantly high. The computational times obtained are summarized in Table 4. Note that no performance is reported for GridLMM since it cannot handle such a large number of variance components (the memory size required for matrix storage—>80Go for the first iteration—becomes highly prohibitive). The table of the variance component estimates is given in S4 Appendix.

**Table 4:** Computational time (in sec.) associated to the analysis of the NAM dataset using Model (3).

gaston	MM4LMM	GEMMA
5207	15739	>30000

Lastly, note that Model (3) assumes a homogeneous error variance across trials, a strong hypothesis that is highly unlikely in practice. The third strategy is then to analyze the full dataset using Model (3), except that one now assumes that $E_{k}∼N\left(0,\sigma_{ET_{k}}^{2}I_{n_{k}}\right)$ for each trial *k*. A comparison between the homogeneous and trial specific error variance models based on the BIC criterion confirms that the heterogeneous error variance model is to be preferred for the NAM dataset (BIC(homogeneous)=15,279, BIC(specific)=14,850, smaller is better). Although highly desirable for the statistical analysis, this last model cannot be fitted by the algorithms presented here except for MM4LMM, that run the analysis in 4410 seconds.

## Discussion

As illustrated in the Results section, the MM algorithm is a competitive algorithm in the context of ReML inference for VC mixed models. When *K* = 2, it can be combined with the simultaneous orthogonalization trick to compete with algorithms dedicated to the 2-component case such as FaST-LMM or BOLT-LMM when applied to datasets of moderate size. For large values of *K* the number of available methods reduces to gaston, GEMMA and MM4LMM, the last one being the more versatile to e.g. handle models including heterogeneous error variances. This versatility is important in the context of GS, GWAS or VCA since it gives access to models more complex than the “reference” model that only includes a polygenic and an error components (as proposed in [10]). Note that the MM4LMM R package also provides estimated standard error values for the variance parameters that help to better interpret the results when the number of variance components is high. These features make MM4LMM a method of choice for i) the analysis of multi-environment trials and ii) the analysis of crossing designs where the modeling of non-additive marker effects is at stake, as these two settings are usually characterized by a large number of variance components.

The analysis of the NAM dataset showed that the MME trick is only beneficial if the number of observations is much higher than the total number of latent effects, but may be detrimental otherwise. Also note that the MME trick requires each matrix *V*_*k*_ in the model to be invertible, a stringent condition that is not required by the ReML procedure itself, and is not satisfied in many applications. For these reasons the use of the MME trick should be restricted to some applications only, an optional strategy that is available in the MM4LMM package but not in most packages/softwares.

## Conclusion

The Min-Max algorithm is a simple alternative to the by-default AI-ReML algorithm that is commonly implemented in most packages. We demonstrated that most classical speed-up tricks used in the context of mixed model inference can be easily combined with the MM algorithm, yielding an efficient estimation procedure than can compete with state of the art competitors in most contexts that are commonly encountered in animal or plant genetics—even those for which efficient alternatives exist (such as the *K* = 2 case). This first study opens a way for new algorithmic developments in the field of VC mixed models and more generally in ReML inference for other classes of mixed models. A limitation for such further developments is the fact that MM methods require the derivation of a specific surrogate function for each class of mixed model to be considered, making the extension of the inference procedure to e.g. auto-regressive or factor analytic models [29] not straightforward.

## Methods

### Variance component mixed model

In this article we focus on variance component models of the form:

$$\begin{array}{c} Y∼N(X\beta ,\sum_{k=1}^{K}\sigma_{k}^{2}V_{k}) \end{array}$$ (4)

where *Y* is a vector of *n* observations, *X* is an incidence matrix, *β* is the vector of fixed effects, *V*_*k*_ is the (known) correlation matrix associated to the *k*^*th*^ random effect and $\gamma =(\sigma_{1}^{2},\ldots ,\sigma_{K}^{2})$ is the vector of variances associated to the *K* random effects. In what follows *X* is assumed to be a full rank matrix. A special case of Model (4) is the following mixed model:

$$\begin{array}{ccc} Y & = & X\beta +\sum_{k=1}^{K-1}Z_{k}U_{k}+E\\ \text{with} & & \left\{ \begin{array}{ccc} U_{k} & ∼N(0,\sigma_{k}^{2}R_{k}),\;\;k=1,\ldots ,K-1, & \\ E & ∼N(0,\sigma_{K}^{2}V_{K}) & \\ U_{1} & ⊥\ldots ⊥U_{K-1}⊥E & , \end{array}\right. \end{array}$$ (5)

where *U*_*k*_ is the *k*^th^ random effect vector of size *n*_*k*_, *Z*_*k*_ (resp. *R*_*k*_) is the incidence matrix (resp. the correlation matrix) associated with random effect *U*_*k*_, *E* is an error vector, and notation *A* ⊥ *B* stands for “*A* and *B* are independent”. Model (5) boils down to Model (4) where $V_{k}=Z_{k}R_{k}Z_{k}^{T}$. Lastly, we introduce $\Sigma_{\gamma }=\sum_{k=1}^{K}\sigma_{k}^{2}V_{k}$, the covariance matrix of vector *Y*.

The goal is to infer the unknown fixed effects and variance parameters *β* and *γ*. Here we consider the Restricted Maximum Likelihood (ReML) estimation procedure [30, 31].

Let *Π*_*X*^⊥^_ = *I* − *X*(*X*^*T*^ *X*)^−1^ *X*^*T*^ be the projection matrix on *span*(*X*)^⊥^, and *M* be any matrix built from the columns of *Π*_*X*^⊥^_ such that *M* is of full rank and *rank*(*M*) = *rank*(*Π*_*X*^⊥^_) = *m*. Applying *M* to the initial data vector *y* allows one to get rid of the fixed effects. The restricted (log-) likelihood corresponds to the (log-) likelihood of the transformed data *My*, and has the following expression (up to a constant):

$$\begin{array}{c} L_{R}\left(\gamma \right)=-\frac{1}{2}[\text{log}(|X^{T}\Sigma_{\gamma }^{-1}X|)+\text{log}(|\Sigma_{\gamma }\left|\right)+y^{T}P_{\gamma }y] \end{array}$$ (6)

where |*H*| stands for the determinant of matrix *H* and

$$\begin{array}{ccc} P_{\gamma } & = & M^{T}{\left(M\Sigma_{\gamma }M^{T}\right)}^{-1}M\\ & = & \Sigma_{\gamma }^{-1}-\Sigma_{\gamma }^{-1}X{\left(X^{T}\Sigma_{\gamma }^{-1}X\right)}^{-1}X^{T}\Sigma_{\gamma }^{-1}. \end{array}$$

Note that $L_{R}\left(\gamma \right)$ does not depend on *β* (since *MX* = 0 by construction), nor on the specific choice of *M* thanks to the second expression of *P*_*γ*_ above [32]. Variance parameters *γ* can be estimated by applying the classical Maximum Likelihood procedure to $L_{R}$, then fixed effects can be obtained using the following formula:

$$\begin{array}{c} \hat{\beta }={\left(X^{T}\Sigma_{\hat{\gamma }}^{-1}X\right)}^{-1}X^{T}\Sigma_{\hat{\gamma }}^{-1}y. \end{array}$$

Although quite popular, the ReML procedure may be quite challenging from a computational point of view, the bottleneck being the maximization of the log-likelihood (6) w.r.t. *γ*. Although the first derivative of $L_{R}$ with respect to $\sigma_{k}^{2}$ has a simple expression:

$$\begin{array}{ccc} \frac{\partial L_{R}}{\partial \sigma_{k}^{2}} & = & -\frac{1}{2}[tr\left(P_{\gamma }V_{k}\right)-y^{T}P_{\gamma }V_{k}P_{\gamma }y], \end{array}$$

solving the *K* equations $\frac{\partial L_{R}}{\partial \sigma_{k}^{2}}=0$, *k* = 1, …, *K* does not lead to a closed form expression for $\hat{\gamma }$. Consequently likelihood maximization has to be performed numerically. The next section presents the Newton optimization algorithm and its derivatives to obtain the ReML variance estimates.

### Newton based algorithms

#### Newton algorithm

Let first rewrite Model (4) as

$$\begin{array}{c} Y=X\beta +ZU+E \end{array}$$

where *Z* = (*Z*_1_|…|*Z*_*K*−1_), $U=(U_{1}^{T}\left|\ldots \right|U_{K-1}^{T}{)}^{T}$. The joint distribution of (*U*, *E*) is

$$\begin{array}{ccc} \left[\begin{array}{c} U\\ E \end{array}\right] & ∼ & N(0,\sigma_{K}^{2}[\begin{array}{cc} G_{\delta } & 0\\ 0 & V_{K} \end{array}])\\ \text{where}\;\;\delta & = & \left(\frac{\sigma_{1}^{2}}{\sigma_{K}^{2}},\ldots \frac{\sigma_{K-1}^{2}}{\sigma_{K}^{2}}\right)\\ \text{and}\;\;G_{\delta } & = & (\begin{array}{cccc} \delta_{1}R_{1} & 0 & \ldots & 0\\ 0 & \delta_{2}R_{2} & \ldots & 0\\ \ldots & \ldots & \ldots & \ldots \\ 0 & 0 & \ldots & \delta_{K-1}R_{K-1} \end{array}). \end{array}$$

The restricted likelihood $L_{R}$ can be reformulated as:

$$\begin{array}{c} \begin{array}{cc} L_{R}\left(\delta ,\sigma_{K}^{2}\right)= & -\frac{1}{2}[m\text{log}\left(\sigma_{K}^{2}\right)+\text{log}(|X^{T}\Sigma_{\delta }^{-1}X\left|\right)\\ & +\text{log}(|\Sigma_{\delta }\left|\right)+\frac{y^{T}P_{\delta }y}{\sigma_{K}^{2}}] \end{array} \end{array}$$

where Σ_*δ*_ = *ZG*_*δ*_ *Z*^*T*^ + *V*_*K*_, $P_{\delta }=\sigma_{K}^{2}P_{\gamma }$ and *m* = *rank*(*M*). Starting from this last expression, one can perform optimization of $L_{R}$ using an iterative scheme like the Newton algorithm that requires the first and second derivatives of $L_{R}$ w.r.t. both *δ* and $\sigma_{K}^{2}$. The first derivatives are

$$\begin{array}{c} \begin{array}{cc} {\left[\nabla L_{R}(\delta ,\sigma_{K}^{2})\right]}_{k} & =-\frac{1}{2}(tr\left(P_{\delta }V_{k}\right)-\frac{y^{T}P_{\delta }V_{k}P_{\delta }y}{\sigma_{K}^{2}})\;\;1\leq k<K,\\ {\left[\nabla L_{R}(\delta ,\sigma_{K}^{2})\right]}_{K} & =-\frac{1}{2}(\frac{m}{\sigma_{K}^{2}}-\frac{y^{T}P_{\delta }y}{\sigma_{K}^{4}})\;. \end{array} \end{array}$$

Similarly, the second derivatives are

$$\begin{array}{c} \begin{array}{cc} {\left[HL_{R}(\delta ,\sigma_{K}^{2})\right]}_{kk^{\prime }} & =\frac{1}{2}tr\left(P_{\delta }V_{k}P_{\delta }V_{k^{\prime }}\right)-\frac{y^{T}P_{\delta }V_{k}P_{\delta }V_{k^{\prime }}P_{\delta }y}{\sigma_{K}^{2}},\\ {\left[HL_{R}(\delta ,\sigma_{K}^{2})\right]}_{kK} & =-\frac{1}{2}\frac{y^{T}P_{\delta }V_{k}P_{\delta }y}{\sigma_{K}^{4}},\\ {\left[HL_{R}(\delta ,\sigma_{K}^{2})\right]}_{KK} & =\frac{m}{2\sigma_{K}^{4}}-\frac{y^{T}P_{\delta }y}{\sigma_{K}^{6}}\;. \end{array} \end{array}$$

Denoting $\nabla_{L_{R}}^{\left(t\right)}$ and $H_{L_{R}}^{\left(t\right)}$ the gradient and the Hessian matrix of $L_{R}$ evaluated at point $\left(\delta^{\left(t\right)},\sigma_{K}^{2(t)}\right)$ respectively, the Newton method then iterates the following recursion:

$$\begin{array}{c} (\begin{array}{c} \delta^{\left(t+1\right)}\\ \sigma_{K}^{2(t+1)} \end{array})=(\begin{array}{c} \delta^{\left(t\right)}\\ \sigma_{K}^{2(t)} \end{array})-{\left[H_{L_{R}}^{\left(t\right)}\right]}^{-1}\nabla_{L_{R}}^{\left(t\right)}\;. \end{array}$$

A classical shortcut consists in making use of the fact that the last gradient component leads to an explicit expression of $\sigma_{K}^{2}$ when the ratio *δ* is known:

$$\begin{array}{c} {\hat{\sigma }}_{K}^{2}\left(\delta \right)=\frac{y^{T}P_{\delta }y}{m}. \end{array}$$ (7)

One can then apply the Newton algorithm to update *δ* only, which reduces the number of unknown parameter by one in the Newton update procedure. This trick is classically known as the “profiling” trick. Additional computational shortcuts are presented in the Computational shortcuts section.

#### Fisher scoring and average information

It has been suggested [11, 30] that the use of alternative matrices in place of the Hessian matrix in the Newton procedure could be beneficial in terms of convergence rate and/or computational burden. The first alternative, known as the Fisher algorithm, consists in replacing $H_{L_{R}}^{\left(t\right)}$ by its expected value. The expectations of the Hessian matrix terms are

$$\begin{array}{c} \begin{array}{cc} E{\left[HL_{R}(\delta ,\sigma_{K}^{2})\right]}_{kk^{\prime }} & =-0.5\times tr(P_{\delta }V_{k}P_{\delta }V_{k^{\prime }}),\\ E{\left[HL_{R}(\delta ,\sigma_{K}^{2})\right]}_{kK} & =-0.5\times tr\left(P_{\delta }V_{k}\right)/\sigma_{K}^{4},\\ E{\left[HL_{R}(\delta ,\sigma_{K}^{2})\right]}_{KK} & =-0.5\times m/\sigma_{K}^{4}\;. \end{array} \end{array}$$

A second alternative is the use of the Average Information (AI) matrix [12]. The AI matrix is defined as the average of the Hessian and its expectation. The efficiency of this strategy leads in the general expression of the resulting matrix. One has

$$\begin{array}{c} \begin{array}{c} AI_{kk^{\prime }}=\frac{y^{T}P_{\delta }V_{k}P_{\delta }V_{k^{\prime }}P_{\delta }y}{2\sigma_{K}^{2}},\\ AI_{kK}\approx \frac{y^{T}P_{\delta }V_{k}P_{\delta }y}{2\sigma_{K}^{4}},\;AI_{KK}=\frac{y^{T}P_{\delta }y}{2\sigma_{K}^{6}}, \end{array} \end{array}$$ (8)

where for the second term the approximation *tr*(*V*_*k*_ *P*_*δ*_) ≈ *y*^*T*^*P*_*δ*_*V*_*k*_*P*_*δ*_*y* is used. Compared with the previous expressions obtained for the Newton and FS algorithms, computing the AI matrix does not involve any trace computation anymore. Note that *P*_*δ*_ is computed at each step using formula

$$\begin{array}{c} P_{\delta }=\Sigma_{\delta }^{-1}-\Sigma_{\delta }^{-1}X{\left(X^{T}\Sigma_{\delta }^{-1}X\right)}^{-1}X^{T}\Sigma_{\delta }^{-1} \end{array}$$

where *δ* and $\sigma_{K}^{2}$ are fixed at their current value.

#### Computational shortcuts

**Simultaneous orthogonalization**. As mentioned in the Newton based algorithms section, the profiling trick reduces the computational complexity by discarding one of the variance component in the update procedure: the numerical optimization only applies to *δ*, $\sigma_{K}^{2}$ being estimated afterwards using its explicit expression (7). When applied to the case where *K* = 2, profiling may be combined to the simultaneous orthogonalization of the two covariance matrices to obtain an even simpler expression of the restricted likelihood. Assuming one of the two matrices (say *V*_2_) is positive definite, then there exist a matrix Λ and a diagonal matrix *D* such that

$$\begin{array}{c} \Lambda V_{1}\Lambda^{T}=D\;\text{and}\;\Lambda V_{2}\Lambda^{T}=I_{n}. \end{array}$$

One can reexpress the restricted log-likelihood associated with Model (4) as a function of $\sigma_{2}^{2}$, *δ* and *D* as follows:

$$\begin{array}{c} \begin{array}{cc} L_{R}\left(\delta ,\sigma_{2}^{2}\right)= & -\frac{1}{2}[m\text{log}\left(\sigma_{2}^{2}\right)+\text{log}(|D\delta +I_{n}\left|\right)\\ & +\text{log}(|{\tilde{X}}^{T}{\left(D\delta +I_{n}\right)}^{-1}\tilde{X}\left|\right)+\frac{1}{\sigma_{2}^{2}}{\tilde{y}}^{T}P_{\delta }\tilde{y}], \end{array} \end{array}$$ (9)

where $\tilde{y}=\Lambda y$ and $\tilde{X}=\Lambda X$. The expression of ${\hat{\sigma }}_{2}^{2}\left(\delta \right)$ can then be plugged back into Eq (9) to obtain a function that depends on *δ* only:

$$\begin{array}{c} \begin{array}{cc} L_{R}\left(\delta \right)= & -\frac{1}{2}[m\text{log}\left({\hat{\sigma }}_{2}^{2}\left(\delta \right)\right)+\text{log}(|D\delta +I_{n}\left|\right)\\ & +\text{log}(|{\tilde{X}}^{T}{\left(D\delta +I_{n}\right)}^{-1}\tilde{X}|)+m]\;. \end{array} \end{array}$$ (10)

This last expression can then be optimized w.r.t. *δ*. This strategy is implemented in the R package gaston where a Newton Raphson algorithm, followed by a Brent algorithm (if the procedure has not already converged) are used for the optimization of (10), and also in FaST-LMM where the optimization is first performed on a grid then refined using the Brent algorithm [7, 9]. One can observe that the simultaneous orthogonalization trick drastically reduces the computational burden whenever many models with identical random effects but different fixed effects have to be adjusted, the orthogonalization being performed only once (i.e. Λ is common to all models).

**Henderson equation shortcut**. As mentioned earlier, one needs to invert matrix Σ_*δ*_ at each step to update matrix *P*_*δ*_. This operation is the computational bottleneck of the optimization procedure and may become cumbersome when the number of measurements *n* is large. In some configurations this step may be relaxed by deriving the quantities required for the update of *δ* from the Henderson Mixed Model Equations (MME)

$$\begin{array}{c} \left[\begin{array}{cc} X^{T}V_{K}^{-1}X & X^{T}V_{K}^{-1}Z\\ Z^{T}V_{K}^{-1}X & Z^{T}V_{K}^{-1}Z+G_{\delta }^{-1} \end{array}][\begin{array}{c} \hat{\beta }\\ \hat{u} \end{array}]=[\begin{array}{c} X^{T}V_{K}^{-1}y\\ Z^{T}V_{K}^{-1}y \end{array}\right] \end{array}$$ (11)

Noting *C* the coefficient matrix appearing in the left hand side of the equation, one first notices that solving the system requires the inversion of *C*, of size ∑_*k*_ *n*_*k*_ + *p*, where *n*_*k*_ is the length of vector *U*_*k*_ and *p* is the rank of matrix *X*. Second, it has been shown that the quantities appearing in AI matrix (8) can be reexpressed using *C*^−1^, details are given in S5 Appendix [12]. One has:

$$\begin{array}{c} \begin{array}{c} AI_{kk^{\prime }}=\frac{\left[y^{T}P_{\delta }Z_{k}\right]R_{k}\left[Z_{k}^{T}P_{\delta }Z_{k^{\prime }}\right]R_{k^{\prime }}\left[Z_{k^{\prime }}P_{\delta }y\right]}{2\sigma_{K}^{2}},\\ AI_{kK}=\frac{\left[y^{T}P_{\delta }Z_{k}\right]R_{k}\left[Z_{k}^{T}P_{\delta }y\right]}{2\sigma_{K}^{4}},\;AI_{KK}=\frac{y^{T}P_{\delta }y}{2\sigma_{K}^{6}} \end{array} \end{array}$$

and

$$\begin{array}{c} \begin{array}{c} Z^{T}P_{\delta }y=G_{\delta }^{-1}\hat{u}\\ Z^{T}P_{\delta }Z=G_{\delta }^{-1}-G_{\delta }^{-1}{\left[C^{-1}\right]}_{uu}G_{\delta }^{-1}\\ P_{\delta }y=V_{K}^{-1}\hat{e}. \end{array} \end{array}$$ (12)

where $\hat{e}=y-X\hat{\beta }-Z\hat{u}$ and [*C*^−1^]_*uu*_ corresponds to the submatrix of *C*^−1^ associated with the random component *u*. Assuming all matrices *V*_*k*_ are invertible (i.e. definite positive), all these expressions are easily obtained from $\hat{\beta }$, $\hat{u}$ and *C*^−1^. As soon as ∑_*k*_ *n*_*k*_ + *p* ≪ *n* it becomes computationally efficient to compute the AI matrix through the MME rather than through direct inversion of *P*_*δ*_. In the following, this shortcut will be referred to as the “MME trick”.

### Min-Max algorithm for ReML

#### MM algorithm for ReML inference

MM algorithms represent another class of iterative schemes [15]. We provide a brief overview of the MM principle based on the previous reference. Consider an optimization problem where one aims at finding the minimizer θ* of a function *f*(*θ*) (in our setting $f=-L_{R}$ and *θ* = *γ*), one builds at each step *t* a surrogate function *g*^(*t*)^ satisfying

$$\begin{array}{c} g^{\left(t\right)}\left(\theta \right)\geq f\left(\theta \right)\;\;\text{and}\;\;g^{\left(t\right)}\left(\theta^{\left(t-1\right)}\right)=f\left(\theta^{\left(t-1\right)}\right)\;, \end{array}$$

where *θ*^(*t*−1)^ is the current evaluation of θ*. Assuming the surrogate function can be minimized easily, one defines

$$\begin{array}{c} \theta^{\left(t\right)}=\text{arg}\underset{\theta }{\text{min}}\;g^{\left(t\right)}\left(\theta \right)\;. \end{array}$$

One can show that the sequence (*θ*^(*t*)^)_*t*≥1_ satisfies the descent property *f*(*θ*^(*t*+1)^) ≤ *f*(*θ*^(*t*)^). In practice, the convergence is assessed using a convergence criterion such as

$$\begin{array}{c} \left|\right|\theta_{t}-\theta_{t-1}{\left|\right|}_{\infty }=\underset{i}{\text{max}}\left(\right|\theta_{t,i}-\theta_{t-1,i}\left|\right)<\epsilon \end{array}$$

or

$$\begin{array}{c} f\left(\theta_{t-1}\right)-f\left(\theta_{t}\right)<\epsilon . \end{array}$$

In the present article both criteria were used with *ϵ* = 10^−5^.

In the context of variance component mixed models, a MM method has been proposed to maximize the likelihood [16]. Following the same line of proof, we present an MM algorithm for ReML inference. The main difficulty to apply MM optimization is to derive the sequence of surrogate functions. Proposition 1 provides the surrogate function at step *t* + 1 for the ReML optimization problem:

**Proposition 1** *Define function g*^(*t*+1)^(*γ*) *as*

$$\begin{array}{ccc} g^{\left(t+1\right)}\left(\gamma \right) & = & \frac{1}{2}\sum_{k=1}^{K}[\sigma_{k}^{2}tr\left(P_{\gamma }^{\left(t\right)}V_{k}\right)+\frac{\sigma_{k}^{4(t)}}{\sigma_{k}^{2}}y^{T}P_{\gamma }^{\left(t\right)}V_{k}P_{\gamma }^{\left(t\right)}y]\\ & & +\text{log}(|M\Sigma_{\gamma }^{\left(t\right)}M^{T}|)-m, \end{array}$$ (13)

*where m* = *rank*(*M*). *Then*

$$\begin{array}{c} g^{\left(t+1\right)}\left(\gamma \right)\geq -L_{R}\left(\gamma \right) \end{array}$$

*where equality holds at point γ*^(*t*)^.

The proof of Proposition 1 is adapted from the one given for ML inference [16] and is given in S6 Appendix. Because at each step *t* the surrogate function *g*^(*t*)^ is linear with respect to $\sigma_{1}^{2},\ldots ,\sigma_{K}^{2}$, one easily obtains its optimizer by setting its gradient at 0. This provides the following update for the variance parameters:

$$\begin{array}{c} \sigma_{k}^{2(t+1)}=\sigma_{k}^{2(t)}\sqrt{\frac{y^{T}P_{\gamma }^{\left(t\right)}V_{k}P_{\gamma }^{\left(t\right)}y}{tr(P_{\gamma }^{\left(t\right)}V_{k})}}\;. \end{array}$$ (14)

The next section presents the adaptation of the computational tricks presented in the Newton based algorithms section to the ReML MM procedure.

#### Computational shortcuts for the ReML MM procedure

**Two matrix shortcut**. In the particular case when *K* = 2 the correlation matrices can be jointly orthogonalized. Similar to the profiling trick, we introduce

$$\begin{array}{c} \begin{array}{cc} g^{\left(t+1\right)}\left(\delta \right)= & \frac{1}{2}[m\;\text{log}\left(\frac{1}{m}{\tilde{y}}^{T}P_{\delta }^{\left(t\right)}\left(\frac{\delta^{2(t)}}{\delta }D+I_{n}\right)P_{\delta }^{\left(t\right)}\tilde{y}\right)\\ & +tr\left(P_{\delta }^{\left(t\right)}\left(D\delta +I\right)\right)+c^{\left(t\right)}]\;, \end{array} \end{array}$$

where *c*(*t*) is an irrelevant constant. As detailed in S7 Appendix, one can show that optimizing function *g*^(*t*+1)^ boils down to solving a quadratic function that admits a unique positive solution corresponding to *δ*^(*t*+1)^.

***K* matrix shortcut**. When relevant, the MME trick can be applied to speed up the computation of the quantities appearing in the surrogate function (13). Since $P_{\delta }=\sigma_{K}^{2}P_{\gamma }$ and $V_{k}=Z_{k}R_{k}Z_{k}^{T}$, the update formulas (14) can be rewritten as follows:

$$\begin{array}{c} \sigma_{k}^{2(t+1)}=\sigma_{k}^{2(t)}\sqrt{\frac{y^{T}P_{\delta }^{\left(t\right)}Z_{k}R_{k}Z_{k}^{T}P_{\delta }^{\left(t\right)}y}{\sigma_{K}^{2(t)}tr\left(Z_{k}^{T}P_{\delta }^{\left(t\right)}Z_{k}R_{k}\right)}} \end{array}$$

and can be computed using expressions (12) for *k* = 1, …, *K* − 1 (recall that *Z* does not include *Z*_*K*_). For the case *k* = *K*, the numerator can be easily obtained from the expression of $P_{\delta }^{\left(t\right)}y$ in (12), and *tr*(*PV*_*K*_) can be calculated using the following proposition:

**Proposition 2** *Define* $S=V_{K}^{-1}-V_{K}^{-1}X{\left(X^{T}V_{K}^{-1}X\right)}^{-1}X^{T}V_{K}^{-1}$.

$$\begin{array}{c} Then\;tr\left(PV_{K}\right)=m-tr\left(Z^{T}SZ{\left[C^{-1}\right]}_{uu}\right). \end{array}$$

Note that *S* does not need any update and can be computed at once. The demonstration is given in S8 Appendix.

**MM Acceleration**. Similar to EM algorithms, MM algorithms can benefit from accelerating strategies to achieve better rates of convergence (by reducing the number of iterations required to achieve a given precision). Here we combined the MM algorithm with a squared iterative method [33]. Assuming one aims at minimizing a function *f* using a MM algorithm, note *θ*^(*t*−2)^, *θ*^(*t*−1)^ and *θ*^(*t*)^ the MM estimates obtained at steps *t* − 2, *t* −1 and *t*, respectively. At step *t* one also computes

$$\begin{array}{ccc} r & = & \theta^{\left(t-1\right)}-\theta^{\left(t-2\right)},\;v=\theta^{\left(t\right)}-\theta^{\left(t-2\right)},\;\alpha =-{\left||r|\right|}_{2}/{\left||v|\right|}_{2}\\ \theta^{\left(t^{\prime }\right)} & = & \theta^{\left(t-2\right)}-2\alpha r+\alpha^{2}v \end{array}$$

If *f*(*θ*^(*t*′)^) < *f*(*θ*^(*t*)^) then *θ*^(*t*)^ ← *θ*^(*t*′)^. The acceleration process is then iterated with *θ*^(*t*+2)^.

## Supporting information

S1 Code Data and Code for Two variance component analyses available as zip file. (ZIP)

S2 Code Data and Code for Four variance component GWAS available as zip file. (ZIP)

S3 Code Data and Code for Four variance component VCA available as zip file. (ZIP)

S1 Appendix Wald test procedure. (PDF)

S2 Appendix Data correction step for the NAM dataset. (PDF)

S3 Appendix Table of variance component estimates for model (2). (PDF)

S4 Appendix Table of variance component estimates for model (3) with common error variance. (PDF)

S5 Appendix Proof of equalities in Eq (12). (PDF)

S6 Appendix MM surrogate function for the ReML procedure. (PDF)

S7 Appendix Two variance component shortcut for the MM algorithm. (PDF)

S8 Appendix Proof of Proposition 2. (PDF)
