# Supplementary material for: Efficient ReML inference in variance component mixed models using a Min-Max algorithm
Source: PLoS Comput Biol. 2022 Jan 24;18(1):e1009659. doi: 10.1371/journal.pcbi.1009659 (PMC8824334; doi:10.1371/journal.pcbi.1009659)
Supplement: S1 Code — (ZIP) [file pcbi.1009659.s001.zip › TwoVarianceComponents/Rmd/SupplementaryResults_TVC.html]

Supplementary Results for the two variance component analysis of the DMY trait


# Supplementary Results for the two variance component analysis of the DMY trait

#### F. Laporte

## Required package

The following packages are required to perform the analysis that follows:

```
suppressMessages(library('gaston'))
library('MM4LMM')
suppressMessages(library(tidyverse))
```

## Load and shape the data

First fix the paths of the Data and Results directories:

```
PathToData <- "./"
PathToResults <- "./"
```

Then specify the trait of interest:

```
Trait <- "DM_Yield_Flo"
```

All data are stored as .RData:

```
## Load genotypes
GenoF <- readRDS(paste0(PathToData,"GenoF.rds"))
## Load phenotypes
PhenoF <- readRDS(paste0(PathToData,"PhenoF.rds"))
## Load the kinship matrix
kinship <- readRDS(paste0(PathToData,"KinF.rds"))
## Load the genetic map
MapF <- readRDS(paste0(PathToData,"MapF.rds"))
```

## Build kinship matrices specific to each chromosome

The IBD kinship matrix may be built from a set of markers based on the following function:

```
ComputeKinship <- function(geno.mat){
  Freq <- rowMeans(geno.mat)/2
  SD <- 2*sqrt(Freq*(1-Freq))
  geno.std <- (geno.mat-2*Freq)/SD
  kinship <- crossprod(geno.std)/length(Freq)
  return(kinship)
}
```

Let build the kinship matrix based on the full set of "PZE" markers, and the different kinship matrices computed by removing all "PZE" markers of a given chromosome:

```
## Get the genotypic matrix for the PZE markers
Geno <- t(GenoF)[str_which(MapF$Name,pattern = "PZE"),]
Info <- MapF[str_which(MapF$Name,pattern = "PZE"),]
Chr.list <- unique(MapF$Chr)

## Kinship matrix, all markers
K.all <- ComputeKinship(Geno)
rownames(K.all) <- colnames(K.all) <- PhenoF$Accession

## Kinship matrix, excluding chromosomes one by one
K.chr <- map(Chr.list, ~{
  Tmp <- Geno[which(Info$Chr!=.x),] %>% ComputeKinship()
  rownames(Tmp) <- colnames(Tmp) <- PhenoF$Accession
  return(Tmp)
  })
```

Let also split the initial genotypic data into different tables corresponding to the different chromosomes:

```
Geno.list <- map(Chr.list,~GenoF[,which(MapF$Chr==.x)])
```

## Perform inference and tests

```
## The methods to be compared
Methods <- c('MM.all','MM.chr','gaston.all','score.all','score.chr')

## Pvalue storage
Pval.df <- matrix(NA,nrow=nrow(MapF),ncol=length(Methods)) %>% 
  as.data.frame()
rownames(Pval.df) <- MapF$Name
colnames(Pval.df) <- Methods

## Time storage
Time <- matrix(NA,1,ncol=length(Methods)) %>% 
  as.data.frame()
colnames(Time) <- Methods
```

Now apply the different procedures:

```
## Specify some options
NbCores <- 1 ## Nb of threads for MM4LMM
setThreadOptions(numThreads=NbCores) ## Nb of threads for gaston
Crit <- 10e-5 ## Precision during inference

## Inference using MM4LMM
ptm <- proc.time()["elapsed"]
Fit.MM.all <- MMEst(Y=PhenoF[,Trait], 
               X = 1/2*GenoF , 
               VarList = list(K.all,diag(1,nrow(K.all))),
               CritLogLik = Crit,
               CritVar = Crit,
               NbCores=NbCores)
Res.MM.all <- AnovaTest(Fit.MM.all,Type="TypeI",NbCores=NbCores)
Time$MM.all <- proc.time()["elapsed"]-ptm

## Inference using gaston and wald test
BED <- as.bed.matrix(GenoF)
ptm <- proc.time()["elapsed"]
Res.gaston.all <- association.test(BED, 
                                   PhenoF[,Trait], 
                                   method="lmm",
                                   response="quantitative",
                                   eigenK=eigen(K.all),
                                   test="wald",
                                   eps=Crit,
                                   verbose=F)
Time$gaston.all <- proc.time()["elapsed"]-ptm

## Inference using score tests
ptm <- proc.time()["elapsed"]
Res.score.all<- association.test(BED, 
                                 PhenoF[,Trait], 
                                 method="lmm",
                                 response="quantitative",
                                 K=K.all,
                                 eigenK=eigen(K.all),
                                 test="score",
                                 eps=Crit,
                                 verbose=F)
Time$score.all <- proc.time()["elapsed"]-ptm

## Inference with FaST-LMMa
# Writing the needed files
FAM_data <- as.data.frame(cbind(1:nrow(GenoC),rownames(GenoC),0,0,0,PhenoC[,Trait]))
names(FAM_data) <- c("famid","id","father","mother","sex","pheno")
BIM_data <- as.data.frame(cbind(MapC[colnames(GenoC),"Chr"],colnames(GenoC),0,MapC[colnames(GenoC),"Pos"],"A","T"))
names(BIM_data) <- c("chr","id","dist","pos","A1","A2")
BED <- as.bed.matrix(GenoC,FAM_data,BIM_data)
write.bed.matrix(BED,basename=paste0(PathToResults,"BEDdata_All"))

DataFast <- as.data.frame(cbind(1:nrow(GenoC),rownames(GenoC),PhenoC[,Trait]))
write.table(DataFast,file=paste0(PathToResults,"PhenoStudied.txt"),quote = FALSE,row.names=F,col.names=F)

var = c("var", paste(seq(1, nrow(geno)), rownames(geno), sep=" "))
kinship_fast = cbind(paste(seq(1, nrow(geno)), rownames(geno), sep=" "), K.all)
kinship_fast = rbind(as.vector(var), kinship_fast)
write.table(kinship_fast,file=paste0(PathToResults,"Kin_All.txt"),row.names = F,col.names=F,quote=F,sep="\t")

# Running the algorithm
path.fast <- ""
command.fastlmm = paste0(path.fast,"fastlmmc -REML -verboseOut -bfile ", PathToResults, "BEDdata_All -pheno ",PathToResults,"PhenoStudied.txt -sim ", PathToResults,"Kin_All.txt -simLearnType Once -out ",PathToResults,"ResFastLmmApprox_All.csv -maxThreads ", NbCores)
ptm <- proc.time()["elapsed"]
Toto <- system(command.fastlmm,intern = TRUE)
Time$FastApprox.all <- proc.time()["elapsed"] - ptm


## Inference using MM4LMM, per chromosome
ptm <- proc.time()["elapsed"]
Res.MM.chr <- map2(Geno.list,K.chr, ~{
  Fit <- MMEst(Y=PhenoF[,Trait], 
        X = 1/2*.x , 
        VarList = list(.y,diag(1,nrow(.y))),
        CritLogLik = Crit,
        CritVar = Crit,
        NbCores=NbCores)
  Res <- AnovaTest(Fit,Type="TypeI",NbCores=NbCores)
})
Time$MM.chr <- proc.time()["elapsed"]-ptm

## Inference using score tests, per chromosome 
BED.chr <- map(Geno.list,as.bed.matrix)
ptm <- proc.time()["elapsed"]
Res.score.chr<- map2(BED.chr,K.chr, ~{
  association.test(.x, 
                   PhenoF[,Trait], 
                   method="lmm",
                   response="quantitative",
                   K=.y,
                   eigenK=eigen(.y),
                   test="score",
                   eps=Crit,
                   verbose=F)
})
Time$score.chr <- proc.time()["elapsed"]-ptm

## Inference using FaST-LMMa
# Writing the needed files
for (i in 1:length(Geno.list)){
  geno <- Geno.list[[i]]
  FAM_data <- as.data.frame(cbind(1:nrow(geno),rownames(geno),0,0,0,PhenoC[,Trait]))
  names(FAM_data) <- c("famid","id","father","mother","sex","pheno")
  BIM_data <- as.data.frame(cbind(MapC[colnames(geno),"Chr"],colnames(geno),0,MapC[colnames(geno),"Pos"],"A","T"))
  names(BIM_data) <- c("chr","id","dist","pos","A1","A2")
  BED <- as.bed.matrix(geno,FAM_data,BIM_data)
  write.bed.matrix(BED,basename=paste0(PathToResults,"BEDdata_Chr",i))
  
  var = c("var", paste(seq(1, nrow(geno)), rownames(geno), sep=" "))
  kinship_fast = cbind(paste(seq(1, nrow(geno)), rownames(geno), sep=" "), K.chr[[i]])
  kinship_fast = rbind(as.vector(var), kinship_fast)
  write.table(kinship_fast,file=paste0(PathToResults,"Kin_Chr",i,".txt"),row.names = F,col.names=F,quote=F,sep="\t")
}

# Running the algorithm
ptm <- proc.time()["elapsed"]
for (i in 1:length(Geno.list)){
  command.fastlmm = paste0(path.fast,"fastlmmc -REML -verboseOut -bfile ", PathToResults, "BEDdata_Chr",i," -pheno ",PathToResults,"PhenoStudied.txt -sim ", PathToResults,"Kin_Chr",i,".txt -simLearnType Once -out ",PathToResults,"ResFastLmmApprox_Chr",i,".csv -maxThreads ", NbCores)
  Toto <- system(command.fastlmm,intern = TRUE)
}
Time$FastApprox.chr <- proc.time()["elapsed"] - ptm
```

## Summary of the results

We first collect the set of pvalues obtained from the different methods and

```
## Collect pvalues
Pval.df$MM.all <- map(Res.MM.all, ~ .x["Xeffect","pval"])
Pval.df$gaston.all <- Res.gaston.all$p
Pval.df$score.all <- Res.score.all$p
Pval.df$MM.chr <- rapply(Res.MM.chr, 
                         f = function(x){x["Xeffect","pval"]})
Pval.df$score.chr <- map(Res.score.chr,~ .x$p) %>% Reduce('c',.)

FileRes <- read.csv(paste0(PathToResults,"ResFastLmmApprox_All.csv"))
Pval_FaST <- pchisq(FileRes$WaldStat,df=1,lower.tail=F)
names(Pval_FaST) <- FileRes$SNP
Pval.df$FastApprox.all <- Pval_FaST[as.character(MapF$Name)]

Pval_FaST_Comb <- c()
for (i in 1:length(Geno.list)){
  FileRes <- read.csv(paste0(PathToResults,"ResFastLmmApprox_Chr",i,".csv"))
  Pval_FaST <- pchisq(FileRes$WaldStat,df=1,lower.tail=F)
  names(Pval_FaST) <- FileRes$SNP
  Pval_FaST_Comb <- c(Pval_FaST_Comb,Pval_FaST)
}
Pval.df$FastApprox.chr <- Pval_FaST_Comb[as.character(MapF$Name)]
```

If you do not infer models using he previous lines you can use the "p-value\_LOCO.rds" file:

```
## Load pvalues in supplementary
Pval.df <- readRDS(paste0(PathToData,"p-value_LOCO.rds"))
```

Identify the associated lists of significant markers at level 5%:

```
## Get the significant marker lists
Qtl.list <- map(Pval.df, ~{
  .x %>% 
    '<='(0.05/3527) %>% 
    which
})

## How many significant markers per method ?
map_int(Qtl.list,length)
```

```
##         MM.all         MM.chr     gaston.all      score.all      score.chr 
##              5              6              5              1              2 
## FastApprox.all FastApprox.chr 
##              2              3
```

```
## Have a look at the significant markers
Qtl.list %>% 
  Reduce('union',.) %>% 
  Pval.df[.,]
```

```
##                     MM.all       MM.chr   gaston.all    score.all    score.chr
## SYN10537      2.492817e-06 1.272157e-06 2.456789e-06 3.917225e-05 3.042399e-05
## SYN10528      2.492817e-06 1.272157e-06 2.456796e-06 3.917225e-05 3.042399e-05
## PZE-101030022 8.601747e-06 4.642735e-06 8.477310e-06 1.054916e-04 8.941580e-05
## PZE-101123079 1.346757e-05 4.822420e-06 1.346822e-05 2.582482e-05 1.150139e-05
## SYN13856      6.386929e-06 2.147820e-06 6.387806e-06 1.357531e-05 5.907612e-06
## PZE-101123102 2.557286e-05 9.754838e-06 2.557638e-05 4.555310e-05 2.144391e-05
##               FastApprox.all FastApprox.chr
## SYN10537        2.177759e-05   1.632779e-05
## SYN10528        2.177759e-05   1.632779e-05
## PZE-101030022   6.662390e-05   5.534995e-05
## PZE-101123079   1.353696e-05   5.330149e-06
## SYN13856        6.458444e-06   2.451344e-06
## PZE-101123102   2.585059e-05   1.093830e-05
```

## Session information

The present results were obtained using the following versions of the packages:

```
sessionInfo()
```

```
## R version 3.6.3 (2020-02-29)
## Platform: x86_64-w64-mingw32/x64 (64-bit)
## Running under: Windows 10 x64 (build 19043)
## 
## Matrix products: default
## 
## locale:
## [1] LC_COLLATE=French_France.1252  LC_CTYPE=French_France.1252   
## [3] LC_MONETARY=French_France.1252 LC_NUMERIC=C                  
## [5] LC_TIME=French_France.1252    
## 
## attached base packages:
## [1] stats     graphics  grDevices utils     datasets  methods   base     
## 
## other attached packages:
##  [1] forcats_0.5.0      stringr_1.4.0      dplyr_1.0.4        purrr_0.3.4       
##  [5] readr_1.4.0        tidyr_1.1.2        tibble_3.0.4       ggplot2_3.3.3     
##  [9] tidyverse_1.3.0    MM4LMM_2.1.0       gaston_1.5.7       RcppParallel_5.0.0
## [13] Rcpp_1.0.5        
## 
## loaded via a namespace (and not attached):
##  [1] tidyselect_1.1.0  xfun_0.19         haven_2.3.1       lattice_0.20-38  
##  [5] colorspace_2.0-0  vctrs_0.3.8       generics_0.1.0    htmltools_0.5.1.1
##  [9] yaml_2.2.1        rlang_0.4.11      pillar_1.4.7      withr_2.4.2      
## [13] glue_1.4.2        DBI_1.1.0         dbplyr_2.1.0      modelr_0.1.8     
## [17] readxl_1.3.1      lifecycle_1.0.0   munsell_0.5.0     gtable_0.3.0     
## [21] cellranger_1.1.0  rvest_0.3.6       evaluate_0.14     knitr_1.30       
## [25] parallel_3.6.3    broom_0.7.6       backports_1.2.1   scales_1.1.1     
## [29] jsonlite_1.7.1    fs_1.5.0          hms_0.5.3         digest_0.6.27    
## [33] stringi_1.5.3     grid_3.6.3        cli_3.0.1         tools_3.6.3      
## [37] magrittr_2.0.1    crayon_1.4.1      pkgconfig_2.0.3   MASS_7.3-51.5    
## [41] ellipsis_0.3.2    Matrix_1.2-18     xml2_1.3.2        reprex_1.0.0     
## [45] lubridate_1.7.9.2 rstudioapi_0.13   assertthat_0.2.1  rmarkdown_2.5    
## [49] httr_1.4.2        R6_2.5.0          compiler_3.6.3
```
