# Supplementary material for: Efficient ReML inference in variance component mixed models using a Min-Max algorithm
Source: PLoS Comput Biol. 2022 Jan 24;18(1):e1009659. doi: 10.1371/journal.pcbi.1009659 (PMC8824334; doi:10.1371/journal.pcbi.1009659)
Supplement: S1 Code — (ZIP) [file pcbi.1009659.s001.zip › TwoVarianceComponents/Rmd/TVC_Analysis.html]

Two variance components: association analysis


# Two variance components: association analysis

#### F. Laporte

## Set the parameters

One first needs to set the following parameters :

- the path of the directory where the data are stored,
- the phenotype under study,
- the number of cored that will be used to run the different algorithms.

```
PathToData <- "./"
PathToResults <- "./"
StudyPheno <- c("DM_Yield_Flo","Tasseling_GDUb6")
NbCores <- 1
```

The required packages are the following ones:

Package versions are provided at the end of this document.

## Load and format the results

The datasets to load are the following:

- GenoF: the genotype file,
- PhenoF the phenotype file,
- KinC: kinship matrix (as used in MM4LMM),
- MapF: the marker information file.
- MapPosGen: Genetic map of markers

```
GenoF <- readRDS(paste0(PathToData,"GenoF.rds"))
PhenoF <- readRDS(paste0(PathToData,"PhenoF.rds"))
kinship <- readRDS(paste0(PathToData,"KinF.rds"))
MapF <- readRDS(paste0(PathToData,"MapF.rds"))
MapPosGen <- readRDS(paste0(PathToData,"GeneticMap.rds"))
```

Let build some matrices to store the results:

```
TablePvalGaston <- matrix(NA,ncol=ncol(MapF)+1,nrow=ncol(PhenoF)-1)
rownames(TablePvalGaston) <- colnames(PhenoF)[-1]
TablePvalMM <- TablePvalGaston

NbQTLGaston <- rep(NA,ncol(PhenoF)-1)
names(NbQTLGaston) <- colnames(PhenoF)[-1]
NbQTLMM <-  TimeMM <- TimeGaston <- TimeLme <- 
  TimeFastLmm <- TimeFastLmmApprox <- NbQTLGaston

## Kinship
rownames(kinship) <- colnames(kinship) <- PhenoF$Accession
# A function to transform a matrix into a positive definite matrix
MP <- function(Mat){
  res <- (1-1/nrow(Mat))*Mat
  diag(res) <- diag(Mat)
  return(res)
}
# kinship matrix transformation into a positive definite matrix. This step is mendatory when using ASReml)
kinship <- MP(kinship)
```

The following codelines must be uncommented if you aim at running FaST-LMM or GEMMA in order to shape the kinship matrix as required by these procedures.

```
## Prepare kinship matrix for FaST-LMM
 var = c("var", paste(seq(1, nrow(GenoF)), rownames(GenoF), sep=" "))
 kinship_fast = cbind(paste(seq(1, nrow(GenoF)), rownames(GenoF), sep=" "), kinship)
 kinship_fast = rbind(as.vector(var), kinship_fast)
 write.table(kinship_fast,file=paste0(PathToResults,"kinship_fast.txt"),
             quote=F,row.names = F,col.names = F,sep="\t")

## Prepare kinship matrix for GEMMA
 write.table(kinship,file=paste0(PathToResults,"kinship_Gemma.txt"),
             quote=F,row.names = F,col.names = F)
```

## GWAS Analysis

In what follows GWAS are performed using **MM4LMM** and **gaston** only.

```
setThreadOptions(numThreads=NbCores)


ResMM <- ResGaston <- ResASReml <- ResGrid <- ResLme <- list()
TimeMM <- TimeGaston <- TimeASReml <- TimeGemma <- 
  TimeBolt <- TimeGrid <- TimeFaST <- TimeFaST_Approx <- 
  TimeLME4 <-c()

Crit <- 10e-5

## Main loop for model fitting
invisible(sapply(StudyPheno , function(trait){

    
    ## Inference using MM4LMM
    ptm <- proc.time()["elapsed"]
    ResMM[[trait]] <<- MMEst(Y=PhenoF[,trait] , X = GenoF , 
                             VarList = list(kinship,diag(1,nrow(kinship))),
                             CritLogLik = Crit, CritVar = Crit,
                             NbCores=NbCores)
    TimeMM[trait] <<- proc.time()["elapsed"]-ptm

  ## Generate BED data using gaston
    FAM_data <- as.data.frame(cbind(1:nrow(GenoF),
                                    rownames(GenoF),
                                    0,0,0,PhenoF[,trait]))
  names(FAM_data) <- c("famid","id","father","mother","sex","pheno")
  BIM_data <- as.data.frame(cbind(MapF[colnames(GenoF),"Chr"],
                              colnames(GenoF),0,
                              MapF[colnames(GenoF),"Pos"],"A","T"))
  names(BIM_data) <- c("chr","id","dist","pos","A1","A2")
    BED <- as.bed.matrix(GenoF,FAM_data,BIM_data)

    ## Inference using gaston
    ptm <- proc.time()["elapsed"]
    ResGaston[[trait]] <<- association.test(BED , PhenoF[,trait] , 
                                            method="lmm" ,response="quanti",
                                            eigenK=eigen(kinship),test="wald")
    TimeGaston[trait] <<- proc.time()["elapsed"]-ptm
}))
```

In order to add GridLMM, Bolt-LMM, FaST-LMM or GEMMA to the study, run the following lines and specify the path to the system command. For BOLT-LMM, FaST-LMM and GEMMA it is also required to save the BED matrix created by gaston (see above). Lastly, be aware that the ASReml procedure is time consuming, and running lme4 is even longer.

```
invisible(sapply(StudyPheno , function(trait){

  # Inference with FaST-LMM
  DataFast <- as.data.frame(cbind(1:nrow(GenoF),rownames(GenoF),PhenoF[,trait]))
  write.table(DataFast,file=paste0(PathToResults,"PhenoStudied.txt"),
              quote = FALSE,row.names=F,col.names=F)
  path.fast <- ""
  command.fastlmm = paste0(path.fast,"fastlmmc -REML -verboseOut -bfile ",
                           PathToResults, "/BEDdata -pheno ", PathToResults,
                           "PhenoStudied.txt -sim ",
                           PathToResults,"kinship_fast.txt -simLearnType Full -out ",
                           PathToResults,"ResFastlmm_",trait,".csv -maxThreads ",
                           NbCores)
  ptm <- proc.time()["elapsed"]
  Toto <- system(command.fastlmm,intern = TRUE)
  TimeFaST[trait] <<- proc.time()["elapsed"] - ptm


    ## Inference using Bolt-LMM
  GenoF_2 <- GenoC[,colnames(GenoF)%in%rownames(MapPosGen)]
  FAM_data <- as.data.frame(cbind(1:nrow(GenoF),rownames(GenoF),0,0,0,PhenoF[,trait]))
  names(FAM_data) <- c("famid","id","father","mother","sex","pheno")
  BIM_data <- as.data.frame(cbind(MapC[colnames(GenoF_2),"Chr"],
                                  colnames(GenoF_2),as.numeric(as.character(MapPosGen$Pos[colnames(GenoF_2)])),
                                  MapF[colnames(GenoF_2),"Pos"],"A","T"))
  names(BIM_data) <- c("chr","id","dist","pos","A1","A2")
  BED <- as.bed.matrix(GenoF_2,FAM_data,BIM_data)
  write.bed.matrix(BED,basename=paste0(PathToResults,"BEDdataBolt"))
  
  # Create LD score regression tabl
  command.ldsc <- paste0("ldsc.py --bfile ",PathToResults,"BEDdataBolt --out ",PathToResults,"/ldsc_output --ld-wind-cm 1")
  Toto <- system(command = command.ldsc)
  
  PhenoBolt <- as.data.frame(cbind(1:nrow(GenoF),rownames(GenoF),PhenoF[,trait]))
  names(PhenoBolt) <- c("FID","IID","pheno")
  write.table(PhenoBolt,file=paste0(PathToResults,"PhenoStudied_Bolt.txt"),
              quote=F,row.names=F)
  path.bolt <- ""
  command.bolt <- paste0(path.bolt,"bolt --bfile=",
                         PathToResults, "BEDdataBolt --phenoFile=", PathToResults,
                         "PhenoStudied_Bolt.txt --phenoCol=pheno --statsFile=",
                         PathToResults,"ResBolt_Flint",trait,".txt --lmm --numThreads=",
                         NbCores," --LDscoresFile=",PathToResults,"/ldsc_output.l2.ldscore.gz 
                         --LDscoresCol L2")
  ptm <- proc.time()["elapsed"]
  Toto <- system(command.bolt,intern = TRUE)
  TimeBolt[trait] <<- proc.time()["elapsed"] - ptm


    ## Inference with Gemma
  path.gemma <- ""
  command.gemma <- paste0("gemma -bfile ",PathToResults, "BEDdata -outdir ",
                          PathToResults," -o ResGemma_Flint", trait,
                          " -lmm 1 -k ", PathToResults,"kinship_Gemma.txt")
  ptm <- proc.time()["elapsed"]
  Toto <- system(command.gemma)
  TimeGemma[trait] <<- proc.time()["elapsed"] - ptm

  ## Inference with GridLMM
  Data <- as.data.frame(PhenoF[,trait])
    names(Data) <- "pheno"
    Marker <- 1/2*GenoF
    rownames(Marker) <- rownames(kinship)
    Data$ID <- rownames(Marker)
    ptm <- proc.time()["elapsed"]
    ResGrid[[trait]] <<- GridLMM_GWAS(formula=pheno~1+(1|ID),test_formula=~1,
                                      reduced_formula=~1, data=Data,X=Marker,
                                      relmat=list(ID=kinship),method="REML",
                                      algorithm="Fast",mc.cores=8)
    TimeGrid[trait] <<- proc.time()["elapsed"]-ptm


    ## Using LME4 (Time consuming)
    myReTrms<-function(ListZ){
    reTrms<-list()
    reTrms$Zt    <- Matrix(t(Reduce('cbind',ListZ)),sparse=TRUE)
    reTrms$theta <- rep(1,length(ListZ))  # Initial Value of the covariance parameters
    reTrms$Lind  <- rep(1:length(ListZ),unlist(lapply(ListZ,ncol))) 
    # an integer vector of indices determining the mapping of the elements 
    # of the theta vector to the "x" slot of Lambdat
    reTrms$Gp    <- as.integer(unname(cumsum(c(0,unlist(lapply(ListZ,ncol))))))
    reTrms$lower   <- rep(0,length(ListZ)) # lower bounds on the covariance parameters
    reTrms$Lambdat <- Matrix(diag(rep(1,sum(unlist(lapply(ListZ,ncol))))),sparse=TRUE) 
    # transpose of the sparse relative covariance factor
    reTrms$Ztlist <- lapply(ListZ,function(Z) Matrix(t(Z),sparse=T))
    reTrms$cnms   <-  as.list(names(ListZ)) ; names(reTrms$cnms)<- names(ListZ)
    # Flist is Not very clean (to say the least... )
    reTrms$flist <- lapply(ListZ,function(Z) {
      flist.factor<- as.factor(colnames(Z)[apply(Z,1,function(x)
        which(rmultinom(n=1,size=1,prob =abs(x)/sum(abs(x)))==1) )]);
      levels(flist.factor)<-colnames(Z); return(flist.factor)}) 
    #NULL 
    # list of grouping factors used in the random-effects terms
    return(reTrms)
  }
  mylmer <- function(Response, X, ListZ,REML = TRUE){
    notNA<-!is.na(Response)       # Get rid of the NA
    Response<- Response[notNA]   # Find another solution about the NA ???
    X <- X[notNA,]
    ListZ <- lapply(ListZ,function(Z) Z<-Z[notNA,])
    fr<-model.frame(Response~.,data.frame(Response=Response,X) )
    reTrms <- myReTrms(ListZ)
    devfun <- mkLmerDevfun(fr, X, reTrms)
    opt <- optimizeLmer(devfun)
    return(mkMerMod(environment(devfun), opt, reTrms, fr = fr))
  }
  EigenK <- eigen(make.positive.definite(kinship))
  Zlme <- EigenK$vectors %*% diag(sqrt(EigenK$values))
  ptm <- proc.time()
    ResLme[[trait]] <<- mclapply(1:ncol(GenoF) , function(x) {
        LME <- mylmer(PhenoD[,trait] , cbind(1,1/2*GenoF[,x]) , list(kinship = Zlme))
        Sigma <- as.data.frame(VarCorr(LME))[,"vcov"]
        Var <- kinship * Sigma[1] + diag(Sigma[2],nrow(kinship))
        X <- cbind(1,1/2*geno[,x])
        VarBeta <- solve(t(X) %*% solve(Var) %*% X)
        Beta <- summary(LME)$coefficients[,1]
        C <- c(0,1)
        Wald <- (C %*% Beta)^2 / (t(C)%*%VarBeta%*%C)
        Pval <- pchisq(Wald,df=1,lower.tail=F)
        return(list(Sigma=Sigma,Beta=Beta,Pval=Pval,VarBeta=VarBeta))
        } ,mc.cores=NbCores)
    TimeLme[trait] <<- proc.time()["elapsed"]-ptm["elapsed"]
    names(ResLme[[trait]]) <<- colnames(GenoF)
}))
```

The next chunk allows one to compute p-value vectorsfor MM4LMM and gaston.

```
ListBase <- as.list(rep(NA,length(StudyPheno)))
names(ListBase) <- StudyPheno
Pval_MM <- Pval_ASR <- Pval_Gaston <- Pval_FaST <- 
  Pval_FaSTApprox <- Pval_Bolt <- Pval_Grid <- Pval_Gemma <- 
  ListBase


invisible(sapply(StudyPheno , function(trait){
  
  ## Using MM4LMM
  Test_MM <- AnovaTest(ResMM[[trait]],NbCores=NbCores)
  Pval_MM[[trait]] <<- sapply(Test_MM, function(x) x["Xeffect","pval"])
  
  ## Using gaston
  Pval_Gaston[[trait]] <<- ResGaston[[trait]]$p
  names(Pval_Gaston[[trait]]) <<- ResGaston[[trait]]$id

  }))
```

To compute the p-values for the other procedures run the following codelines:

```
invisible(sapply(StudyPheno , function(trait){
  
  # Using FaST-LMMe (we compute here the chi2 wald statistic)
  FileRes <- read.csv(paste0(PathToResults,"ResFastlmm_",trait,".csv"))
  Pval_FaST[[trait]] <<- pchisq(FileRes$WaldStat,df=1,lower.tail=F)
  names(Pval_FaST[[trait]]) <<- FileRes$SNP
  
  # Using Bolt-LMM (we compute here the chi2 wald statistic)
  FileRes <- read.table(paste0(PathToResults,"ResBolt_Flint",trait,".txt"),
                        sep="\t",header=T)
  Pval_Bolt[[trait]] <<- pchisq(FileRes$BETA**2/FileRes$SE**2,df=1,lower.tail=F)
  names(Pval_Bolt[[trait]]) <<- FileRes$SNP
  
  # Using Gemma (we compute here the chi2 wald statistic)
  FileRes <- read.table(paste0(PathToResults,"ResGemma_Flint",trait,".assoc.txt"),
                        sep="\t",header=T)
  Pval_Gemma[[trait]] <<- pchisq(FileRes$beta**2/FileRes$se**2,df=1,lower.tail=F)
  names(Pval_Gemma[[trait]]) <<- FileRes$rs
  
  # Using GridLMM (we compute here the chi2 wald statistic)
  Pval_Grid[[trait]] <<- pchisq(ResGrid[[trait]]$results$F.1,df=1,lower.tail=F)
  names(Pval_Grid[[trait]]) <<- ResGrid[[trait]]$results$X_ID
  
  # Using LME4
  Pval_LME4[[trait]] <<- sapply(ResLme[[trait]] , function(x) x$Pval)
  names(Pval_LME4[[trait]]) <<- names(ResLme[[trait]])
}))
```

## Load results from the folder

You can directly load the results from the folder:

```
Pval_MM <- readRDS(paste0(PathToResults,"Pval_MM.rds"))
Pval_Gaston <- readRDS(paste0(PathToResults,"Pval_Gaston.rds"))
Pval_FaST <- readRDS(paste0(PathToResults,"Pval_FaST.rds"))
Pval_Bolt <- readRDS(paste0(PathToResults,"Pval_Bolt.rds"))
Pval_Gemma <- readRDS(paste0(PathToResults,"Pval_Gemma.rds"))
Pval_Grid <- readRDS(paste0(PathToResults,"Pval_Grid.rds"))
Pval_LME4 <- readRDS(paste0(PathToResults,"/pvalLME4.rds"))
```

## Display tables and figures

The computational times can be sumarized into the following table, that corresponds to Table 3 in the article:

```
Table <- cbind(TimeGaston,TimeMM,TimeFaST,TimeBolt,TimeGemma,TimeGrid,TimeGrid_Fast,TimeLME4
               )
colnames(Table) <- c("gaston","MM4LMM","FaST-LMM","Bolt-LMM","Gemma","GridLMM","lme4")
print(Table)
```

You can also load the time table within the results folder:

```
Table <- readRDS(paste0(PathToResults,"TableAllTime.rds"))
print(Table)
```

```
##                 gaston MM4LMM FaST-LMM Bolt-LMM  Gemma GridLMM
## DM_Yield_Flo     3.081  6.131   28.210   11.654 14.921   8.616
## Tasseling_GDUb6  5.047 17.175   28.243  325.210 14.889   4.779
```

Generating Figures 1 and 2 of the article requires the association results obtained when using **lme4**, that are time consuming to produce. Here we directly upload the results of the **lme4** analysis as saved in the pvalLME4.rds file.

```
## Loading the p-values obtained by LME4
NumberOfSnp <- 100
for (trait in StudyPheno){
  pvalMMOrd <- names(sort(Pval_MM[[trait]]))
  pvalGastonOrd <- names(sort(Pval_Gaston[[trait]]))
  pvalFastLmmOrd <- names(sort(Pval_FaST[[trait]]))
  pvalBoltOrd <- names(sort(Pval_Bolt[[trait]]))
  pvalGemmaOrd <- names(sort(Pval_Gemma[[trait]]))
  pvalGridOrd <- names(sort(Pval_Grid[[trait]]))
  pvalLmeOrd <- names(sort(Pval_LME4[[trait]]))
  
  PropMM_Lme <- PropGaston_Lme <- PropFastApprox_Lme <- 
    PropFast_Lme <- PropLme_ASR <- PropBolt_Lme <- 
    PropGemma_Lme <- PropGrid_Lme <- rep(NA,NumberOfSnp)
  
  invisible(sapply(1:NumberOfSnp , function(t) {
    PropMM_Lme[t] <<- sum(pvalMMOrd[1:t]%in%pvalLmeOrd[1:t])/t
    PropGaston_Lme[t] <<- sum(pvalGastonOrd[1:t]%in%pvalLmeOrd[1:t])/t
    PropBolt_Lme[t] <<- sum(pvalBoltOrd[1:t]%in%pvalLmeOrd[1:t])/t
    PropGemma_Lme[t] <<- sum(pvalGemmaOrd[1:t]%in%pvalLmeOrd[1:t])/t
    PropGrid_Lme[t] <<- sum(pvalGridOrd[1:t]%in%pvalLmeOrd[1:t])/t
    PropFast_Lme[t] <<- sum(pvalFastLmmOrd[1:t]%in%pvalLmeOrd[1:t])/t
  }))
  
  MinAll <- min(PropBolt_Lme,PropMM_Lme,PropGaston_Lme,
                PropGemma_Lme,PropGrid_Lme,PropFast_Lme,
                PropFastApprox_Lme,PropLme_ASR,na.rm=T)
  plot(PropMM_Lme,type='l', 
       main="Proportion of common markers in the t first p-value",
       xlab="Number of markers considered", ylab="Frequencies", 
       ylim=c(MinAll,1.01),cex.axis=1.5,cex.lab=1.5,
       cex.main=1.5,lwd=2,col=2)
  lines(PropFast_Lme+0.002,col=3,lwd=2)
  lines(PropGaston_Lme-0.002,col=4,lwd=2)       
  lines(PropBolt_Lme,col=5,lwd=2)       
  lines(PropGemma_Lme+0.004,col=6,lwd=2)    
  lines(PropGrid_Lme-0.004,col=7,lwd=2) 
  legend("bottomright",legend=c("MM4LMM","FaST-LMM","gaston","BOLT-LMM","GEMMA","GridLMM"), 
         fill=2:7,cex=1.2,bg="transparent")
}
```

Here is the code to obtain Table 4, without the circles and stars (circles and star s can be obtained by first applying function **p.adjust** to each p-values list then selecting a nominal level to identify the signicant markers).

```
## Select the SNP detected by at least an algorithm with the 5% Gao thershold specified in Rincent et al. (3527 independant tests)
gao_threshold <- 0.05/3527
TableSignif <- list()
for (trait in StudyPheno){
  NamesSnp <- names(Pval_Gaston[[trait]])
  TableAllPval <- cbind(Pval_Gaston[[trait]][NamesSnp],Pval_MM[[trait]][NamesSnp],Pval_FaST[[trait]][NamesSnp],Pval_Bolt[[trait]][NamesSnp],Pval_Gemma[[trait]][NamesSnp],Pval_Grid[[trait]][NamesSnp],Pval_LME4[[trait]][NamesSnp]
                        )
  colnames(TableAllPval) <- c("gaston","MM4LMM","FaST-LMM","Bolt-LMM","Gemma","GridLMM","LME4")
  rownames(TableAllPval) <- NamesSnp
  
  IndDetected <- which(TableAllPval<gao_threshold,arr.ind=T)
  TableSignif[[trait]] <- -log10(TableAllPval[unique(IndDetected[,"row"]),])
  
  print(trait)
  print(TableSignif[[trait]])
}
```

```
## [1] "DM_Yield_Flo"
##                 gaston   MM4LMM FaST-LMM Bolt-LMM    Gemma  GridLMM     LME4
## SYN10537      5.609632 5.603310 5.609632 4.389751 5.609631 5.624691 5.609851
## SYN10528      5.609631 5.603310 5.609632 4.389751 5.609631 5.624691 5.609851
## PZE-101030022 5.071742 5.065413 5.071742 3.968256 5.071744 5.055522 5.071902
## PZE-101123079 4.870690 4.870711 4.870693 5.259025 4.870696 4.870916 4.870834
## SYN13856      5.194648 5.194708 5.194648 5.589391 5.194653 5.193630 5.194902
## PZE-101123102 4.592161 4.592221 4.592161 5.039570 4.592162 4.591191 4.591772
## [1] "Tasseling_GDUb6"
##                 gaston   MM4LMM FaST-LMM Bolt-LMM    Gemma GridLMM     LME4
## PZE-101070781 5.371361 5.371677 5.371350 4.610667 5.371365 5.35110 5.369781
## SYN300        4.443268 4.440217 4.443263 5.223012 4.443268 4.44124 4.447208
```

## Session information

The present results were obtained using the following versions of the packages:

```
sessionInfo()
```

```
## R version 3.6.3 (2020-02-29)
## Platform: x86_64-w64-mingw32/x64 (64-bit)
## Running under: Windows 10 x64 (build 19043)
## 
## Matrix products: default
## 
## locale:
## [1] LC_COLLATE=French_France.1252  LC_CTYPE=French_France.1252   
## [3] LC_MONETARY=French_France.1252 LC_NUMERIC=C                  
## [5] LC_TIME=French_France.1252    
## 
## attached base packages:
## [1] stats     graphics  grDevices utils     datasets  methods   base     
## 
## other attached packages:
## [1] xtable_1.8-4       MM4LMM_2.1.0       gaston_1.5.7       RcppParallel_5.0.0
## [5] Rcpp_1.0.5        
## 
## loaded via a namespace (and not attached):
##  [1] lattice_0.20-38   digest_0.6.27     MASS_7.3-51.5     grid_3.6.3       
##  [5] magrittr_2.0.1    evaluate_0.14     rlang_0.4.11      stringi_1.5.3    
##  [9] Matrix_1.2-18     rmarkdown_2.5     tools_3.6.3       stringr_1.4.0    
## [13] xfun_0.19         yaml_2.2.1        parallel_3.6.3    compiler_3.6.3   
## [17] htmltools_0.5.1.1 knitr_1.30
```
