# Supplementary material for: Efficient ReML inference in variance component mixed models using a Min-Max algorithm
Source: PLoS Comput Biol. 2022 Jan 24;18(1):e1009659. doi: 10.1371/journal.pcbi.1009659 (PMC8824334; doi:10.1371/journal.pcbi.1009659)
Supplement: S2 Code — (ZIP) [file pcbi.1009659.s002.zip › FourVarianceComponents_GWAS/Rmd/FactorialAnalysis.html]

Factorial dataset analysis


# Factorial dataset analysis

#### F. Laporte

## Set the parameters

One first needs to set the following parameters:

- the path to the directory where the data are stored,
- the path to the directory where the results will be stored.

```
RepRes <- "./"
RepData <- "./"
```

The required packages are the following ones:

```
suppressMessages(library(MM4LMM))
suppressMessages(library(Matrix))
suppressMessages(library(gaston))
# suppressMessages(library(GridLMM))
```

Package versions are provided at the end of this document.

## Load and format the data

The datasets to load are the following: - Data\_Facto: the data with all information concerning hybrids (trial, ancestors, phenotypes) - KinF\_Facto: kinship for flint lines inferred using moment method - KinD\_Facto: kinship for dent lines inferred using moment method - doubleRelatedness\_Facto: double relatedness matrix calculated as the multiplication of the parent lines kinship for each couple of hybrids - Geno\_Facto: genotype matrix for the factorial analysis.

```
Data <- readRDS(paste0(RepData,"Data_Facto.rds"))
KinF_NotPD <- readRDS(paste0(RepData,"KinF_Facto.rds"))
KinD_NotPD <- readRDS(paste0(RepData,"KinD_Facto.rds"))
doubleRelatedness_NotPD <- readRDS(paste0(RepData,"doubleRelatedness_Facto.rds"))
Geno <- readRDS(paste0(RepData,"Geno_Facto.rds"))
```

To ensure that matrices are positive definite, a simple diagonal boost is performed:

```
MP <- function(mat){
  N <- nrow(mat)
  Res <- (1-1/N)*mat + 1/N*diag(diag(mat))
  return(Res)
}
KinF <- MP(KinF_NotPD)
KinD <- MP(KinD_NotPD)
doubleRelatedness <- MP(doubleRelatedness_NotPD)
```

Only polymorph markers are kept:

```
SnpPolymorph <- which(apply(Geno,2,function(x) length(unique(x)))>1)
GenoHyb <- Geno[,SnpPolymorph]
```

## Variance component analysis

Here is the code to perform GWAS with MM4LMM and gaston. Be aware that this code is time consuming.

```
KinDComp <- Gd[as.character(HybData$dent),as.character(HybData$dent)]
KinFComp <- Gf[as.character(HybData$flint),as.character(HybData$flint)]


## Inference with MM4LMM
ptm <- proc.time()
ResMM <- MMEst(Y = HybData$GM , Cofactor=matrix(1,ncol=1,nrow=nrow(HybData)) , X = as.matrix(GenoHyb) , VarList=list(Dent=KinDComp , Flint=KinFComp , Crois=doubleRelatedness , Error=diag(1,nrow(HybData))) , CritVar=1e-5 , CritLogLik = 1e-5, Method="Reml" , NbCores=8)
TimeMM <- proc.time()-ptm

## Inference with gaston
ptm <- proc.time()
ResGaston <- mclapply(1:ncol(GenoHyb) , function(x) {
  
  Gaston <- lmm.aireml(Y=HybData$GM, X=cbind(1,GenoHyb[,x]),K=list(KinDComp , KinFComp , doubleRelatedness),verbose=F)
  
  return(list(Beta=Gaston$BLUP_beta , Var = c(Gaston$tau,Gaston$sigma2) , VarBeta = Gaston$varbeta))
},mc.cores=8)
TimeGaston <- proc.time()-ptm
names(ResGaston) <- colnames(GenoHyb)
```

Here is the code to perform GWAS with GridLMM adn GEMMA. GEMMA has to write matrices on your coputer and also Covariate files for each marker.

```
## Inference with GridLMM
Data <- HybData
Data$hybrid <- as.factor(as.character(Data$hybrid))
Data$flint <- as.factor(as.character(Data$flint))
Data$dent <- as.factor(as.character(Data$dent))
Data$ID <- Data$hybrid
ptm <- proc.time()
ResGrid <- GridLMM_GWAS(formula=GM~1+(1|dent)+(1|flint)+(1|hybrid),test_formula=~1,reduced_formula=~1,data=Data,X=as.matrix(GenoHyb),relmat=list(dent=KinD,flint=KinF,hybrid=doubleRelatedness),method="REML",algorithm="Fast",mc.cores=NbCores)
TimeGrid <- proc.time()-ptm

## Inference with GEMMA
write.table(GdComp,file=paste0(RepRes,"/MatGd_Gemma.txt"),quote=F,row.names = F,col.names=F)
write.table(GfComp,file=paste0(RepRes,"/MatGf_Gemma.txt"),quote=F,row.names = F,col.names=F)
write.table(S,file=paste0(RepRes,"/MatS_Gemma.txt"),quote=F,row.names = F,col.names=F)
Path <- rbind(paste0("/home/gqms/flaporte/ArticleFastMixedModel/TechnowData/Results/Mat",c("Gd","Gf","S"),"_Gemma.txt"))
write.table(t(Path),file=paste0(RepRes,"/MatPath_Gemma.txt"),quote=F,row.names = F,col.names=F)

write.table(cbind(HybData$GM),file=paste0(RepRes,"PhenoForGemma.txt"),row.names=F,col.names=F,quote=F)

ptm <- proc.time()
invisible(mclapply(1:ncol(GenoHyb) , function(x) {
  Cof <- cbind(1,GenoHyb[,x])
  write.table(Cof,file=paste0(RepRes,"/Cof_",x,".txt"),col.names=F,row.names=F,quote=F)
  path.gemma <- ""
  command.gemma <- paste0("gemma -p ",RepRes, "PhenoForGemma.txt -c ",RepRes,"Cofactor_Gemma/Cof_",x,".txt -outdir ",RepRes," -o ResGemma_",x," -mk ",RepRes,"MatPath_Gemma.txt -n 1 -vc 2")
  Toto <- system(command.gemma)
},mc.cores=NbCores))
TimeGemma <- (proc.time()-ptm)["elapsed"]
```

## Plotting results

GEMMA run for more than 12 hours. Its results were not considered here. First you can either load our results or use those obtained by the previous lines. To avoid huge computational time to calculate p-value with gaston analysis (calculate and solve the variance matrix for each marker), we saved directly the p-values:

```
TimeMM <- readRDS(paste0(RepRes,"TimeMM_Facto.rds"))
TimeGaston <- readRDS(paste0(RepRes,"TimeGaston_Facto.rds"))
TimeGrid <- readRDS(paste0(RepRes,"TimeGrid_Facto.rds"))

Pval_MM <- readRDS(paste0(RepRes,"Pval_MM_Facto.rds"))
Pval_Grid <- readRDS(paste0(RepRes,"Pval_Grid_Facto.rds"))
Pval_Gaston <- readRDS(paste0(RepRes,"Pval_Gaston_Facto.rds"))
```

If you want to Compute p-values from your own analysis, you can use this lines:

```
## P-values for MM4LMM
Test <- AnovaTest(ResMM)
Pval_MM <- sapply(Test, function(x) {
  if ("Xeffect" %in% rownames(x)){
    return(x["Xeffect","pval"])
  }else{
    return(NA)
  }
})

## P-values for GridLMM
Pval_Grid <- pchisq(ResGrid$results$F.1,df=1,lower.tail=F)
names(Pval_Grid) <- ResGrid$results$X_ID

## P-values For gaston (Time consuming)
Pval_Gaston <- sapply(names(ResGaston), function(x) {
    print(which(names(ResGaston)==x))
    res <- ResGaston[[x]]
    X <- cbind(1,GenoHyb[,x])
    Sig <- KinDComp*res$Var[1]+KinFComp*res$Var[2]+doubleRelatedness*res$Var[3]+diag(res$Var[4],nrow(GenoHyb))
    VarBeta <- solve(crossprod(X,solve(Sig,X)))
    return(pchisq((res$Beta[2]**2)/VarBeta[2,2],df=1,lower.tail=F))
})
names(Pval_Gaston) <- names(ResGaston)
```

Then computational time can be displayed using:

```
TableTime <- c(TimeMM["elapsed"],TimeGaston["elapsed"],TimeGrid["elapsed"])
names(TableTime) <- c("MM4LMM","gaston","GridLMM")
print(TableTime)
```

```
##    MM4LMM    gaston   GridLMM 
## 12421.827 43100.092   107.382
```

And the figure for the p-values can be plotted:

```
SnpNames <- intersect(names(Pval_MM)[!is.na(Pval_MM)],names(Pval_Grid))
par(mar=c(5,5.2,3,1)+.1)
plot(-log10(Pval_Gaston[SnpNames]),-log10(Pval_Grid[SnpNames]),pch=3,col='blue',xlab="gaston -log10(p-value)",ylab="-log10(p-value)",cex.lab=1.7,cex.axis=1.7,xlim=c(0,max(-log10(Pval_Gaston))),ylim=c(0,max(-log10(Pval_Gaston))))
points(-log10(Pval_Gaston[SnpNames]),-log10(Pval_MM[SnpNames]),pch=3,col='purple')
abline(a=0,b=1)
legend("bottomright",legend=c("MM4LMM","GridLMM"),fill=c('purple','blue'))
```

## Session information

The present results were obtained using the following versions of the packages:

```
sessionInfo()
```

```
## R version 3.6.3 (2020-02-29)
## Platform: x86_64-w64-mingw32/x64 (64-bit)
## Running under: Windows 10 x64 (build 19043)
## 
## Matrix products: default
## 
## locale:
## [1] LC_COLLATE=French_France.1252  LC_CTYPE=French_France.1252   
## [3] LC_MONETARY=French_France.1252 LC_NUMERIC=C                  
## [5] LC_TIME=French_France.1252    
## 
## attached base packages:
## [1] stats     graphics  grDevices utils     datasets  methods   base     
## 
## other attached packages:
## [1] gaston_1.5.7       RcppParallel_5.0.0 Rcpp_1.0.5         Matrix_1.2-18     
## [5] MM4LMM_2.1.0      
## 
## loaded via a namespace (and not attached):
##  [1] lattice_0.20-38   digest_0.6.27     MASS_7.3-51.5     grid_3.6.3       
##  [5] magrittr_2.0.1    evaluate_0.14     rlang_0.4.11      stringi_1.5.3    
##  [9] rmarkdown_2.5     tools_3.6.3       stringr_1.4.0     xfun_0.19        
## [13] yaml_2.2.1        parallel_3.6.3    compiler_3.6.3    htmltools_0.5.1.1
## [17] knitr_1.30
```
