# Supplementary material for: Efficient ReML inference in variance component mixed models using a Min-Max algorithm
Source: PLoS Comput Biol. 2022 Jan 24;18(1):e1009659. doi: 10.1371/journal.pcbi.1009659 (PMC8824334; doi:10.1371/journal.pcbi.1009659)
Supplement: S1 Appendix — (PDF) [file pcbi.1009659.s004.pdf]

S1 Appendix: Wald test procedure

The present annex describes the Wald test procedure used throughout the article. Recall that

$$\widehat{\beta} \sim \mathcal{N}\left(\beta, (X^T\Sigma^{-1}X)^{-1}\right),$$

and consequently

$$C\widehat{\beta} \sim \mathcal{N}\left(C\beta, C(X^T\Sigma^{-1}X)^{-1}C^T\right)$$

for any contrast matrix C. The H_0 hypothesis $\{C\beta = 0\}$ can then be tested using the following test statistic:

$$\left(C\widehat{\beta}\right)^T \left[C\left(X^T\Sigma^{-1}X\right)^{-1}C^T\right]^{-1} C\widehat{\beta}$$

that follows a $\chi(r_C)$ distribution under H_0, where r_C is the rank of contrast matrix C. Since the true matrix Σ is unknown one uses its estimated counterpart $\Sigma(\hat{\gamma})$ where the estimated variance parameters are plugged in. The impact of the plug-in procedure on the H_0 distribution is ignored, so that one assumes that

$$\left(C\widehat{\beta}\right)^T \left[C\left(X^T\Sigma(\hat{\gamma})^{-1}X\right)^{-1}C^T\right]^{-1} C\widehat{\beta} \overset{H_0}{\sim} \chi(r_C).$$

Alternatively one can account for the impact of plug-in by using the Kenward Rogers procedure to evaluate approximate degrees of freedom for the denominator - the ratio being now assumed to follow a Fisher distribution, see [**?**] for details. The Kenward Rogers procedure is available in the MM4LMM package.

References

[1] Kenward M, et al. Small sample inference for fixed effects from restricted maximum likelihood. Biometrics. 1997;983–997.
