# Supplementary material for: Efficient ReML inference in variance component mixed models using a Min-Max algorithm
Source: PLoS Comput Biol. 2022 Jan 24;18(1):e1009659. doi: 10.1371/journal.pcbi.1009659 (PMC8824334; doi:10.1371/journal.pcbi.1009659)
Supplement: S2 Appendix — (PDF) [file pcbi.1009659.s005.pdf]

S2 Appendix: Data correction step for the NAM dataset

For each trial the correction model was

$$
\begin{aligned}
&Y = 1\mu + Z_F G_F + Z_D G_D + Z_H G_H + Z_{row} G_{row} + Z_{col} G_{col} + E \\
&G_F \sim \mathcal{N}(0, \sigma_F^2 K_F) \\
&G_D \sim \mathcal{N}(0, \sigma_D^2 K_D) \\
&G_H \sim \mathcal{N}(0, \Phi) \\
&G_{row} \sim \mathcal{N}(0, I_{n_r}) \\
&G_{col} \sim \mathcal{N}(0, I_{n_c}) \\
&E \sim \mathcal{N}(0, I_{n_k}) \\
&G_D \perp G_F \perp G_H \perp G_{row} \perp G_{col} \perp E
\end{aligned}
$$

with Y the phenotypic vector, μ the intercept, G_F, G_D, G_H, G_{row} and G_{col} the random effects corresponding respectively to the Flint, Dent, hybrid, row and column effects, and Z_F, Z_D, Z_H, Z_{row} and Z_{col} their associated incidence matrices. Here n_k (resp. n_{r_k} and n_{c_k}) is the number of measurements (resp. numbers of rows and columns). Matrices K_F, K_D and Φ are defined as in the main document. The corrected phenotypes are then obtained as:

$$Y_{\text{cor}} = Y - (Z_{row} G_{row} + Z_{col} G_{col})$$
