# Supplementary material for: Efficient ReML inference in variance component mixed models using a Min-Max algorithm
Source: PLoS Comput Biol. 2022 Jan 24;18(1):e1009659. doi: 10.1371/journal.pcbi.1009659 (PMC8824334; doi:10.1371/journal.pcbi.1009659)
Supplement: S3 Appendix — (PDF) [file pcbi.1009659.s006.pdf]

S3 Appendix : Table of variance component estimates obtained for model (2).

The considered phenotype is DMY. Standard errors, when available, are given between parenthesis.

Study	VC	gaston	MM4LMM	GEMMA	GridLMM
NAM-8	σ_D^2	0.88	0.88 (0.15)	0.88 (0.15)	0.86
	σ_F^2	0.59	0.59 (0.10)	0.59 (0.10)	0.59
	σ_H^2	0.33	0.33 (0.10)	0.33 (0.10)	0.36
	σ_E^2	2.81	2.81 (0.05)	2.81 (0.05)	2.81
