# Supplementary material for: Efficient ReML inference in variance component mixed models using a Min-Max algorithm
Source: PLoS Comput Biol. 2022 Jan 24;18(1):e1009659. doi: 10.1371/journal.pcbi.1009659 (PMC8824334; doi:10.1371/journal.pcbi.1009659)
Supplement: S4 Appendix — (PDF) [file pcbi.1009659.s007.pdf]

S4 Appendix : Table of variance component estimates for model (3) with common error variance.

The considered phenotype is DMY. Standard errors are given between parenthesis.

VC	gaston	MM4LMM
$sigma_{D}^{2}$	6.58e-01	6.58e-01 (1.05e-01)
$sigma_{F}^{2}$	1.07e+00	1.07e+00 (1.73e-01)
$sigma_{H}^{2}$	5.75e-01	5.75e-01 (1.36e-01)
$sigma_{D,Trial1}^{2}$	1.03e-02	1.03e-02 (2.94e-02)
$sigma_{D,Trial2}^{2}$	4.44e-02	4.44e-02 (3.52e-02)
$sigma_{D,Trial3}^{2}$	5.06e-02	5.07e-02 (5.79e-02)
$sigma_{D,Trial4}^{2}$	2.55e-01	2.55e-01 (1.02e-01)
$sigma_{D,Trial5}^{2}$	7.46e-01	7.46e-01 (2.19e-01)
$sigma_{D,Trial6}^{2}$	1.34e-02	1.34e-02 (3.11e-02)
$sigma_{D,Trial7}^{2}$	5.70e-01	5.70e-01 (1.92e-01)
$sigma_{D,Trial8}^{2}$	9.33e-01	9.33e-01 (2.66e-01)
$sigma_{F,Trial1}^{2}$	1.82e-02	1.82e-02 (3.42e-02)
$sigma_{F,Trial2}^{2}$	1.00e-06	1.67e-07 (3.59e-02)
$sigma_{F,Trial3}^{2}$	1.54e-01	1.54e-01 (9.19e-02)
$sigma_{F,Trial4}^{2}$	4.07e-02	4.08e-02 (5.51e-02)
$sigma_{F,Trial5}^{2}$	7.97e-01	7.97e-01 (2.19e-01)
$sigma_{F,Trial6}^{2}$	1.79e-03	1.81e-03 (2.71e-02)
$sigma_{F,Trial7}^{2}$	4.65e-01	4.65e-01 (1.58e-01)
$sigma_{F,Trial8}^{2}$	8.12e-01	8.13e-01 (2.35e-01)
$sigma_{H,Trial1}^{2}$	1.00e-06	1.05e-08 (1.46e-01)
$sigma_{H,Trial2}^{2}$	1.00e-06	2.89e-08 (1.43e-01)
$sigma_{H,Trial3}^{2}$	2.55e-01	2.55e-01 (1.90e-01)
$sigma_{H,Trial4}^{2}$	1.86e-01	1.86e-01 (1.76e-01)
$sigma_{H,Trial5}^{2}$	4.34e-01	4.34e-01 (2.75e-01)
$sigma_{H,Trial6}^{2}$	1.00e-06	2.12e-07 (1.09e-01)
$sigma_{H,Trial7}^{2}$	4.21e-02	4.53e-02 (1.88e-01)
$sigma_{H,Trial8}^{2}$	1.24e+00	1.24e+00 (4.28e-01)
$sigma_{row,Trial1}^{2}$	1.75e-01	1.75e-01 (5.58e-02)
$sigma_{row,Trial2}^{2}$	4.48e-01	4.48e-01 (1.78e-01)
$sigma_{row,Trial3}^{2}$	1.79e-01	1.79e-01 (7.97e-02)
$sigma_{row,Trial4}^{2}$	1.71e-01	1.71e-01 (5.71e-02)
$sigma_{row,Trial5}^{2}$	1.97e-01	1.97e-01 (7.42e-02)
$sigma_{row,Trial6}^{2}$	4.79e-02	4.79e-02 (3.30e-02)
$sigma_{row,Trial7}^{2}$	2.65e-01	2.65e-01 (7.66e-02)
$sigma_{row,Trial8}^{2}$	5.60e-01	5.60e-01 (1.86e-01)
$sigma_{col,Trial1}^{2}$	1.72e-01	1.72e-01 (7.98e-02)
$sigma_{col,Trial2}^{2}$	9.42e-02	9.42e-02 (4.61e-02)
$sigma_{col,Trial3}^{2}$	4.32e-02	4.32e-02 (3.64e-02)
$sigma_{col,Trial4}^{2}$	7.96e-01	7.96e-01 (2.73e-01)
$sigma_{col,Trial5}^{2}$	1.44e-01	1.44e-01 (5.78e-02)
$sigma_{col,Trial6}^{2}$	2.66e-01	2.66e-01 (1.01e-01)
$sigma_{col,Trial7}^{2}$	1.11e-01	1.11e-01 (5.23e-02)
$sigma_{col,Trial8}^{2}$	1.10e-01	1.10e-01 (5.14e-02)
$sigma_{E}^{2}$	1.87e+00	1.87e+00 (4.15e-02)
