# Supplementary material for: Efficient ReML inference in variance component mixed models using a Min-Max algorithm
Source: PLoS Comput Biol. 2022 Jan 24;18(1):e1009659. doi: 10.1371/journal.pcbi.1009659 (PMC8824334; doi:10.1371/journal.pcbi.1009659)
Supplement: S5 Appendix — (PDF) [file pcbi.1009659.s008.pdf]

S5 Appendix: Proof of equalities in Equation (12)

In this appendix, we present the proof of the 3 equalities in Equation (9) of Section Computational shortcuts. In the following we make extensive use of the Woodbury identity

$$(A + CBC^T)^{-1} = A^{-1} - A^{-1}C(C^T A^{-1} C + B^{-1})^{-1} C^T A^{-1}$$

and the Searle identity

$$A - A(A + B)^{-1} A = B - B(A + B)^{-1} B.$$

To obtain the 3 equalities one first needs to prove the following lemma:

Lemma 1 *Let* $S = V_K^{-1} - V_K^{-1} X (X^T V_K^{-1} X)^{-1} X^T V_K^{-1}$. *Then,*

$$P_\delta = S - SZ(Z^T SZ + G_\delta^{-1})^{-1} Z^T S \tag{1}$$

Proof
Step 1 One can provide the expressions of some basic quantities that will appear in the block inversion of matrix C. The Schur complement of C_{uu} in C can be reformulated as:

$$\overline{C}_{\beta\beta} = X^T \Sigma_\delta^{-1} X$$

One also note that $\overline{C}_{\beta\beta}^{-1} = \left[C^{-1}\right]_{\beta\beta}$.
Similarly, the Schur complement of $C_{\beta\beta} = X^T V_K^{-1} X$ is

$$\overline{C}_{uu} = Z^T SZ + G_\delta^{-1} \ .$$

Step 2 Applying the Woodbury identity to the expression of Σ_δ^{-1} yields

$$\begin{aligned} \Sigma_\delta^{-1} &= \left(V_K + ZG_\delta Z^T\right)^{-1} V_K^{-1} - V_K^{-1} Z (Z^T V_K^{-1} Z + G_\delta^{-1})^{-1} Z^T V_K^{-1} \\ &= \left(V_K + ZG_\delta Z^T\right)^{-1} V_K^{-1} - V_K^{-1} Z C_{uu}^{-1} Z^T V_K^{-1} \end{aligned}$$

This leads to the following expression of P_δ:

$$
\begin{aligned}
P_\delta &= V_K^{-1} - V_K^{-1} Z C_{uu}^{-1} Z^T V_K^{-1} \\
&\quad - \left[V_K^{-1} - V_K^{-1} Z C_{uu}^{-1} Z^T V_K^{-1}\right] X \overline{C}_{\beta\beta}^{-1} X^T \times \\
&\quad \left[V_K^{-1} - V_K^{-1} Z C_{uu}^{-1} Z^T V_K^{-1}\right] \\
&= V_K^{-1} - V_K^{-1} Z C_{uu}^{-1} Z^T V_K^{-1} - V_K^{-1} X \overline{C}_{\beta\beta}^{-1} X^T V_K^{-1} \\
&\quad + V_K^{-1} X \overline{C}_{\beta\beta}^{-1} X^T V_K^{-1} Z C_{uu}^{-1} Z^T V_K^{-1} \\
&\quad + V_K^{-1} Z C_{uu}^{-1} Z^T V_K^{-1} X \overline{C}_{\beta\beta}^{-1} X^T V_K^{-1} \\
&\quad - V_K^{-1} Z C_{uu}^{-1} Z^T V_K^{-1} X \overline{C}_{\beta\beta}^{-1} X^T \times \\
&\quad V_K^{-1} Z C_{uu}^{-1} Z^T V_K^{-1} \\
&= V_K^{-1} - V_K^{-1} \Big\{ X \overline{C}_{\beta\beta}^{-1} X^T \\
&\quad - X \overline{C}_{\beta\beta}^{-1} X^T V_K^{-1} Z C_{uu}^{-1} Z^T - Z C_{uu}^{-1} Z^T V_K^{-1} X \overline{C}_{\beta\beta}^{-1} X^T \\
&\quad + Z C_{uu}^{-1} Z^T + Z C_{uu}^{-1} Z^T V_K^{-1} X \overline{C}_{\beta\beta}^{-1} X^T V_K^{-1} Z C_{uu}^{-1} Z^T \Big\} V_K^{-1} \; . \\
&= V_K^{-1} + V_K^{-1} \Big\{ X \overline{C}_{\beta\beta}^{-1} X^T \\
&\quad X \overline{C}_{\beta\beta}^{-1} C_{\beta u} C_{uu}^{-1} Z^T + Z C_{uu}^{-1} C_{u\beta} \overline{C}_{\beta\beta}^{-1} X^T \\
&\quad - Z \left(C_{uu}^{-1} - C_{uu}^{-1} C_{u\beta} \overline{C}_{\beta\beta}^{-1} C_{\beta u} C_{uu}^{-1} \right) Z^T \Big\} V_K^{-1} \; . \\
&= V_K^{-1} - V_K^{-1} \Big\{ X \left[C^{-1}\right]_{\beta\beta} X^T - X \left[C^{-1}\right]_{\beta u} Z^T - Z \left[C^{-1}\right]_{u\beta} X^T \\
&\quad + Z \left[C^{-1}\right]_{uu} Z^T \Big\} V_K^{-1}.
\end{aligned}
$$

which leads to an expression of P_δ as a function of the blocks of matrix C^{-1}. The process can now be reversed using the same blocks expressed as functions of $C_{\beta\beta}^{-1}$ and $\overline{C}_{uu}^{-1}$:

$$
\begin{aligned}
P_\delta &= V_K^{-1} - \left\{ V_K^{-1}X[C^{-1}]_{\beta\beta}X^TV_K^{-1} + V_K^{-1}X[C^{-1}]_{\beta u}Z^TV_K^{-1} \right. \\
&\quad \left. + V_K^{-1}Z[C^{-1}]_{u\beta}X^TV_K^{-1} + V_K^{-1}Z[C^{-1}]_{uu}Z^TV_K^{-1} \right\} \\
&= V_K^{-1} - V_K^{-1}X\left[(X^TV_K^{-1}X)^{-1} + (X^TV_K^{-1}X)^{-1}\times \right. \\
&\quad \left. X^TV_K^{-1}Z\overline{C}_{uu}^{-1}Z^TV_K^{-1}X(X^TV_K^{-1}X)^{-1}\right]X^TV_K^{-1} \\
&\quad + V_K^{-1}X(X^TV_K^{-1}X)^{-1}X^TV_K^{-1}Z\overline{C}_{uu}^{-1}Z^TV_K^{-1} \\
&\quad + V_K^{-1}Z\overline{C}_{uu}^{-1}Z^TV_K^{-1}X(X^TV_K^{-1}X)^{-1}X^TV_K^{-1} \\
&\quad - V_K^{-1}Z\overline{C}_{uu}^{-1}Z^TV_K^{-1} \\
&= S - V_K^{-1}X(X^TV_K^{-1}X)^{-1}\times \\
&\quad X^TV_K^{-1}Z\overline{C}_{uu}^{-1}Z^TV_K^{-1}X(X^TV_K^{-1}X)^{-1}X^TV_K^{-1} \\
&\quad + V_K^{-1}Z\overline{C}_{uu}^{-1}Z^TV_K^{-1}X(X^TV_K^{-1}X)^{-1}X^TV_K^{-1} \\
&\quad + V_K^{-1}X(X^TV_K^{-1}X)^{-1}X^TV_K^{-1}Z\overline{C}_{uu}^{-1}Z^TV_K^{-1} \\
&\quad - V_K^{-1}Z\overline{C}_{uu}^{-1}Z^TV_K^{-1} \\
&= S + SZ^T\overline{C}_{uu}^{-1}Z^TV_K^{-1}X(X^TV_K^{-1}X)^{-1}X^TV_K^{-1} \\
&\quad - SZ^T\overline{C}_{uu}^{-1}Z^TV_K^{-1} \\
&= S - SZ^T\overline{C}_{uu}^{-1}Z^TS
\end{aligned}
$$

Replacing $\overline{C}_{uu}^{-1}$ by its expression given in step 1 concludes the proof.

Combining the Searle identity with Lemma 1, one easily obtains

$$
\begin{aligned}
Z^TP_\delta Z &= G_\delta^{-1} - G_\delta^{-1}(Z^TSZ + G_\delta^{-1})^{-1}G_\delta^{-1} \\
Z^TP_\delta Z &= G_\delta^{-1} - G_\delta^{-1}[C^{-1}]_{uu}G_\delta^{-1}
\end{aligned}
$$

which provides the second equality of Equation (11).

Using the Schur complement $\overline{C}_{uu}$, one can demonstrate:

$$
\hat{u} = (Z^TSZ + G_\delta^{-1})^{-1}Z^TSy \tag{2}
$$

Proof The MME equation gives the following result:

$$
\begin{aligned}
\hat{u} &= [C^{-1}]_{u\beta}X^TV_K^{-1}y + [C^{-1}]_{uu}Z^TV_K^{-1}y \\
&= -\overline{C}_{uu}^{-1}Z^TV_K^{-1}X(X^TV_K^{-1}X)^{-1}X^TV_K^{-1}y + \overline{C}_{uu}^{-1}Z^TV_K^{-1}y \\
&= \overline{C}_{uu}^{-1}Z^T\left[V_K^{-1} - V_K^{-1}X(X^TV_K^{-1}X)^{-1}X^TV_K^{-1}\right]y \\
&= (Z^TSZ + G_\delta^{-1})^{-1}Z^TSy
\end{aligned}
$$

using the fact that $\overline{C}_{uu} = (Z^TSZ + G_\delta^{-1})$.

Derived from (1) and (2), one has:

$$Z^T P_\delta y = G_\delta^{-1} \hat{u}$$

And derived from (1), (2) and (10) in Section Computational shortcuts of the article, one has:

$$P_\delta y = Sy - SZ\tilde{u} = S(y - X\widehat{\beta} - Z\tilde{u}) = V_K^{-1}\hat{e} \tag{3}$$

where $\hat{e} = y - X\hat{\beta} - Z\hat{u}$.

Proof Using equation (2) and the fact that $SX = 0$:

$$\begin{aligned} P_\delta y &= Sy - SZ(Z^T SZ + G^{-1})^{-1} Z^T Sy \\ &= Sy - SZ\hat{u} \\ &= S(y - X\hat{\beta} - Z\hat{u}) \end{aligned}$$

Then one can demonstrate that $S\hat{e} = V_K^{-1}\hat{e}$, using (10):

$$\begin{aligned} V_K S\hat{e} &= y - Z\hat{u} - X(X^T V_K^{-1} X)^{-1} X^T V_K^{-1} y \\ &\quad + X(X^T V_K^{-1} X)^{-1} X^T V_K^{-1} Z\hat{u} \\ &= y - Z\hat{u} - X(X^T V_K^{-1} X)^{-1} X^T V_K^{-1} y \\ &\quad + X(X^T V_K^{-1} X)^{-1} X^T V_K^{-1} Z \times \\ &\quad \Big\{ -\overline{C}_{uu}^{-1} Z^T V_K^{-1} X (X^T V_K^{-1} X)^{-1} X^T V_K^{-1} y \\ &\quad + \overline{C}_{uu}^{-1} Z^T V_K^{-1} y \Big\} \\ &= y - Z\hat{u} - X[C^{-1}]_{\beta\beta} X^T V_K^{-1} y \\ &\quad - X[C^{-1}]_{\beta u} Z^T V_K^{-1} y \\ &= \hat{e} \end{aligned}$$
