# Supplementary material for: Efficient ReML inference in variance component mixed models using a Min-Max algorithm
Source: PLoS Comput Biol. 2022 Jan 24;18(1):e1009659. doi: 10.1371/journal.pcbi.1009659 (PMC8824334; doi:10.1371/journal.pcbi.1009659)
Supplement: S6 Appendix — (PDF) [file pcbi.1009659.s009.pdf]

S6 Appendix: MM surrogate function for the ReML procedure

In this appendix we prove that the function $g^{(t)}$ introduced in Section MM algorithm for ReML inference satisfies at each step t

$$g^{(t)}(\gamma) \geq -\mathcal{L}_R(\gamma)$$
$$g^{(t)}(\gamma^{(t-1)}) = -\mathcal{L}_R(\gamma^{(t-1)})$$

The proof follows the same lines as the one of [1] for the ML procedure, and is based on following two lemmas:

Lemma 1

$$\log(|A|) \quad \leq \quad \log(|B|) + tr\left[B^{-1}(A-B)\right]$$

with equality when $\gamma = \gamma^{(t)}$

Lemma 2 *(a) The matrix fractional function* $f(A,B) = A^T B^{-1} A$ *is jointly convex in the* $m \times n$ *matrix* A *and the* $m \times m$ *positive definite matrix* B*. (b) The log determinant function* $f(B) = \log(|B|)$ *is concave on the set of positive definite matrices.*

The proof of these two lemmas can be found in [1]. One also needs to prove the following Lemma:

Lemma 3

$$P_\gamma \preceq P_\gamma^{(t)} \sum_{k=1}^{K} \frac{\sigma_k^{4(t)}}{\sigma_k^2} V_k P_\gamma^{(t)}$$

with equality when $\gamma = \gamma^{(t)}$.

proof

$$
\begin{aligned}
& (M\Sigma_\gamma^{(t)}M^T)(M\Sigma_\gamma M^T)^{-1}(M\Sigma_\gamma^{(t)}M^T) \\
= & \left(\sum_{k=1}^K MV_kM^T\sigma_k^{2(t)}\right)\left(\sum_{k=1}^K MV_kM^T\sigma_k^2\right)^{-1}\left(\sum_{k=1}^K MV_kM^T\sigma_k^{2(t)}\right) \\
= & \left(\sum_{k=1}^K \frac{\sigma_k^{2(t)}}{\sum_{j=1}^K \sigma_j^{2(t)}}\frac{\sum_{j=1}^K \sigma_j^{2(t)}}{\sigma_k^{2(t)}}MV_kM^T\sigma_k^{2(t)}\right) \\
& \left(\sum_{k=1}^K \frac{\sigma_k^{2(t)}}{\sum_{j=1}^K \sigma_j^{2(t)}}\frac{\sum_{j=1}^K \sigma_j^{2(t)}}{\sigma_k^{2(t)}}MV_kM^T\sigma_k^{2}\right)^{-1} \\
& \left(\sum_{k=1}^K \frac{\sigma_k^{2(t)}}{\sum_{j=1}^K \sigma_j^{2(t)}}\frac{\sum_{j=1}^K \sigma_j^{2(t)}}{\sigma_k^{2(t)}}MV_kM^T\sigma_k^{2(t)}\right) \\
\preceq & \sum_{k=1}^K \frac{\sigma_k^{2(t)}}{\sum_{j=1}^K \sigma_j^{2(t)}}\left(\frac{\sum_{j=1}^K \sigma_j^{2(t)}}{\sigma_k^{2(t)}}MV_kM^T\sigma_k^{2(t)}\right) \\
& \left(\frac{\sum_{j=1}^K \sigma_j^{2(t)}}{\sigma_k^{2(t)}}MV_kM^T\sigma_k^{2}\right)^{-1}\left(\frac{\sum_{j=1}^K \sigma_j^{2(t)}}{\sigma_k^{2(t)}}MV_kM^T\sigma_k^{2(t)}\right) \\
= & \sum_{k=1}^K \frac{\sigma_k^{4(t)}}{\sigma_k^2}(MV_kM^T)(MV_kM^T)^{-1}(MV_kM^T) \\
= & M\left(\sum_{k=1}^K \frac{\sigma_k^{4(t)}}{\sigma_k^2}V_k\right)M^T
\end{aligned}
$$

That leads to:

$$
\begin{aligned}
(M\Sigma_\gamma M^T)^{-1} & \preceq (M\Sigma_\gamma^{(t)}M^T)^{-1}M\left(\sum_{k=1}^K \frac{\sigma_k^{4(t)}}{\sigma_k^2}V_k\right)M^T \\
& \quad (M\Sigma_\gamma^{(t)}M^T)^{-1} \\
M^T(M\Sigma_\gamma M^T)^{-1}M & \preceq M^T(M\Sigma_\gamma^{(t)}M^T)^{-1}M\left(\sum_{k=1}^K \frac{\sigma_k^{4(t)}}{\sigma_k^2}V_k\right)M^T \\
& \quad (M\Sigma_\gamma^{(t)}M^T)^{-1}M \\
P_\gamma & \preceq P_\gamma^{(t)}\left(\sum_{k=1}^K \frac{\sigma_k^{4(t)}}{\sigma_k^2}V_k\right)P_\gamma^{(t)}
\end{aligned}
$$

Applying the last inequality to $\gamma = \gamma^{(t)}$ yields an equality.

The function $-\mathcal{L}_R(\gamma)$ can be expressed as:

$$-\mathcal{L}_R(\gamma) = \frac{1}{2}\left[\log(|M\Sigma_\gamma M^T|) + y^TP_\gamma y\right]$$

Using the two previous lemmas, the inequality is verified:

$$\begin{aligned}
-\mathcal{L}_R(\gamma) &\leq \frac{1}{2}\Big[\log(|M\Sigma_\gamma^{(t)}M^T|) \\
&\quad + tr\Big[(M\Sigma_\gamma^{(t)}M^T)^{-1}(M\Sigma_\gamma M^T - M\Sigma_\gamma^{(t)}M^T)\Big] \\
&\quad + y^T P_\gamma^{(t)}(\sum_{k=1}^{K}\frac{\sigma_k^{4(t)}}{\sigma_k^2}V_k)P_\gamma^{(t)}y\Big] \\
-\mathcal{L}_R(\gamma) &\leq g(\gamma|\gamma^{(t)})
\end{aligned}$$

References

[1] Zhou H, et al. MM algorithms for variance components models. Journal of Computational and Graphical Statistics. 2019;28(2);350–361.
