# Supplementary material for: Efficient ReML inference in variance component mixed models using a Min-Max algorithm
Source: PLoS Comput Biol. 2022 Jan 24;18(1):e1009659. doi: 10.1371/journal.pcbi.1009659 (PMC8824334; doi:10.1371/journal.pcbi.1009659)
Supplement: S7 Appendix — (PDF) [file pcbi.1009659.s010.pdf]

S7 Appendix: Two variance component shortcut for the MM algorithm

This section provides the details of the derivation of the quadratic form of the surrogate function in the case where the mixed model has only two variance components. After using the profiling trick and the simultaneous orthogonalization of the covariance matrices, the restricted log-likelihood writes

$$-\mathcal{L}_R(\delta|\tilde{y}) = \frac{1}{2}\left[m\log(\hat{\sigma}_2^2(\delta)) + \log(|M(D\delta + I_n)M^T|) + m\right] ,$$

where $\hat{\sigma}_2^2(\delta) = \frac{1}{m}y^T P_\delta y$. Applying lemma 4 in S6 Appendix gives the following upper bound for $\hat{\sigma}_2^2(\delta)$:

$$\frac{1}{m}y^T P_\delta^{(t)}(D\frac{\delta^{2(t)}}{\delta} + I_n)P_\delta^{(t)} y \ .$$

From lemma 3 in S6 Appendix, $\log(|M(D\delta + I_n)M^T|)$ can be upper bounded by:

$$\begin{aligned}\log(|M(D\delta^{(t)} + I_n)M^T|) \quad & +tr[(M(D\delta^{(t)} + I_n)M^T)^{-1} \\ & (M(D\delta + I_n)M^T - M(D\delta^{(t)} + I_n)M^T)] \ .\end{aligned}$$

Combining the two upper bounds yields the surrogate function:

$$\begin{aligned} g^{(t)}(\delta) &= \frac{1}{2}\{m\log\left(\frac{1}{m}\tilde{y}^T P_\delta^{(t)}\left(\frac{\delta^{2(t)}}{\delta}D + I_n\right)P_\delta^{(t)}\tilde{y}\right) \\ &\quad +tr(P_\delta^{(t)}(D\delta + I)) - m\} \\ &= \frac{1}{2}\left\{m\log\left(\left[\frac{1}{m}\tilde{y}^T P_\delta^{(t)}\delta^{2(t)}DP_\delta^{(t)}\tilde{y}\right]\times\frac{1}{\delta} + \left[\frac{1}{m}\tilde{y}^T P_\delta^{(t)}P_\delta^{(t)}\tilde{y}\right]\right)\right. \\ &\quad \left. + \left[tr(P_\delta^{(t)}D)\right]\times\delta + \left[tr(P_\delta^{(t)}) - m\right]\right\} \\ &= \frac{1}{2}\left\{m\log\left(\frac{B}{\delta} + A\right) + C\delta + \lambda\right\} \end{aligned}$$

where A, B, C and λ are coefficients that depend on $\delta^{(t)}$ but not on δ. The first derivative of the surrogate function is then

$$\frac{dg^{(t)}(\delta)}{d\delta} = \frac{1}{2}\left[\frac{-mB}{B\delta + A\delta^2} + C\right]$$
