# Supplementary material for: Efficient ReML inference in variance component mixed models using a Min-Max algorithm
Source: PLoS Comput Biol. 2022 Jan 24;18(1):e1009659. doi: 10.1371/journal.pcbi.1009659 (PMC8824334; doi:10.1371/journal.pcbi.1009659)
Supplement: S8 Appendix — (PDF) [file pcbi.1009659.s011.pdf]

S8 Appendix: Proof of Proposition 2

First note that from the definition of S one can easily check that

$$tr(SV_K) = m \quad \text{and} \quad SV_K S = S.$$

Then, from Equation (1) in S2 Appendix, one has

$$\begin{aligned} tr(P_\delta V_K) &= tr(SV_K) - tr\left(SZ\left(Z^T SZ + G_\delta^{-1}\right) Z^T SV_K\right) \\ &= m - tr\left(Z^T SV_K SZ\left(Z^T SZ + G_\delta^{-1}\right)\right) \\ &= m - tr\left(Z^T SZ\left(Z^T SZ + G_\delta^{-1}\right)\right) \\ &= m - tr\left(Z^T SZ\left[C^{-1}\right]_{uu}\right) \ . \end{aligned}$$
